# Unraveling the sequence-dependent polymorphic behavior of d(CpG) steps in B-DNA

**DOI:** 10.1093/nar/gku809

**Published:** 2014-09-15

**Authors:** Pablo Daniel Dans, Ignacio Faustino, Federica Battistini, Krystyna Zakrzewska, Richard Lavery, Modesto Orozco

**Affiliations:** 1 Joint BSC-CRG-IRB Research Program in Computational Biology, Institute for Research in Biomedicine (IRB Barcelona), Baldiri Reixac 10, Barcelona 08028, Spain; 2 Bases Moléculaires et Structurales des Systèmes Infectieux, Univ. Lyon I/CNRS UMR 5086, IBCP, 7 Passage du Vercors, Lyon 69367, France; 3 Departament de Bioquimica, Facultat de Biologia, Avgda Diagonal 647, Barcelona 08028, Spain

## Abstract

We have made a detailed study of one of the most surprising sources of polymorphism in B-DNA: the high twist/low twist (HT/LT) conformational change in the d(CpG) base pair step. Using extensive computations, complemented with database analysis, we were able to characterize the twist polymorphism in the d(CpG) step in all the possible tetranucleotide environment. We found that twist polymorphism is coupled with BI/BII transitions, and, quite surprisingly, with slide polymorphism in the neighboring step. Unexpectedly, the penetration of cations into the minor groove of the d(CpG) step seems to be the key element in promoting twist transitions. The tetranucleotide environment also plays an important role in the sequence-dependent d(CpG) polymorphism. In this connection, we have detected a previously unexplored intramolecular C-H···O hydrogen bond interaction that stabilizes the low twist state when 3′-purines flank the d(CpG) step. This work explains a coupled mechanism involving several apparently uncorrelated conformational transitions that has only been partially inferred by earlier experimental or theoretical studies. Our results provide a complete description of twist polymorphism in d(CpG) steps and a detailed picture of the molecular choreography associated with this conformational change.

## INTRODUCTION

The highly polymorphic nature of the DNA molecule has been known since the fifties, when researchers realized that changes in the solvent composition could lead to conformational transitions in DNA resulting in very different X-ray diffraction patterns (1). Half a century later, a more complete picture of DNA structural polymorphism in double, triple and quadruple helical conformations has emerged (2–8). However, it is only in the last decade that the availability of high-resolution X-ray and NMR data has revealed DNA polymorphism at the molecular level in different sequence contexts. This polymorphism is evident even for double-stranded DNA oligomers that adopt an overall physiological B-form (9,10). Recent systematic database analysis (11) has presented clear experimental evidence that sequence strongly influences the equilibrium conformation of DNA, and made it clear that sequence-averaged helical parameters used in nearest-neighbor models (12–15) are only a rough approximation of true duplex conformations. Recent atomistic molecular dynamics (MD) simulations have complemented available experimental information, allowing us to obtain structural information on base pair steps in sequence contexts where little or no experimental data exists (11). In particular, the efforts of the Ascona B-DNA (ABC) consortium have been very useful in providing information on the conformational properties of the 10 unique base pairs steps surrounded by all the unique flanking base pairs (constituting the 136 unique tetranucleotide sequences) (16–18). For comparison, a recent analysis of the crystallographic data in the nucleic acid database (19) (using a resolution cutoff of 2.5 Å and limiting the analysis to isolated, unmodified B-DNA oligomers), shows that almost half of the tetranucleotides are not present among the resolved structures.

The ABC results highlighted two unexpected facts: (i) the importance of nearest-neighbors in determining the geometries of base pair steps (i.e. the need to consider tetranucleotide effects) and (ii) the existence of a surprising polymorphism for specific base pair steps, even within a given tetranucleotide environment (18). Such structural polymorphism became evident from multi-nanosecond MD simulations, where instead of the usual normal distributions, non-Gaussian distributions reflected the existence of two rapidly interconverting states for some helical parameters in certain steps (11,19). Analysis of a large number of trajectories revealed that d(YpR) steps (or simply YR steps), especially TA and CG, show the largest tendency to display binormal distributions in helical parameters (11). The scarcity of experimental data makes it difficult to confirm most cases of binormality emerging from MD simulations, with the exception of the twist distribution in the CG step. In this particular case, convincing experimental evidence (11) confirms that binormality is not a force-field artifact. It is consequently important to understand this property of CG steps, how it can influence recognition (20–22) and what role it could play in defining regulatory regions in DNA (23–26).

A statistical study of CG twist distributions from a large ensemble of MD simulations detected binormality in all the possible flanking base pair environments (10 unique tetranucleotide sequences, given the inversion symmetry of the CG step). In 8 cases out of 10, the two equilibrium twist values were clearly distinct and statistically meaningful (11). The corresponding conformational states can be divided into a ‘low twist’ (LT) population, with an average value of around 20°, and a ‘high twist’ (HT) population, with a twist around 40°. LT↔HT transitions occur on the picosecond time scale, but convergence of the twist populations is reached only after ∼300 ns, suggesting that these transitions are coupled with other slower conformational changes. Furthermore, the LT/HT ratio is strongly dependent on the tetranucleotide environment, suggesting that the CG sequence context is likely to be important in biological processes such as the recognition by intercalators (27,28), the nucleosome wrapping (29–31) or interactions with regulatory proteins (20–22).

Here we make a detailed investigation of the mechanics of LT↔HT transitions for DNA duplexes containing CG steps in the 10 unique tetranucleotide environments by carrying out and analyzing a set of long atomistic MD trajectories, simulated in the presence of explicit waters and physiological salt concentrations (K^+^Cl^−^ or Na^+^Cl^−^). The information from these simulations was combined with quantum mechanical calculations in order to decipher the mechanics of the LT↔HT transitions. These transitions, which are tightly coupled with nearby BI/BII conversions in the phosphodiester backbone (18), also turn out to depend on cation dynamics in both thermodynamic and kinetic terms. Lastly, we discuss the possible biological implications of CG polymorphism by making links between structural and genomic data.

## MATERIALS AND METHODS

### Molecular dynamics simulations

#### Unrestrained MD simulations

Ten 12-mer DNA duplexes of sequence CGCG**X**CG**Y**CGCG, with X and Y selected to represent all the 10 possible unique tetranucleotide environments containing a central CG step, were each simulated for 0.5 μs. Starting structures were taken from Arnott canonical B-DNA (32), and oligomers were built using the Nucleic Acid Builder (33). The oligomers were simulated using periodic boundary conditions with a truncated octahedral box and an explicit solvent environment consisting of TIP3P water molecules (34), with a minimum thickness of 11 Å around the solute. The DNA net charge was neutralized with K^+^ or Na^+^ cations and K^+^Cl^−^ or Na^+^Cl^−^ ion pairs were added to reach a concentration of ∼0.15 M. Counterions were initially placed randomly, at a minimum distance of 5 Å from the solute and 3.5 Å from one another. Considering two salt environments led to a total of 20 simulations and more than 10 μs of unrestrained trajectories. An additional very long 4 μs trajectory of Dickerson dodecamer (with Na^+^ as cation) was analyzed to check for potential convergence issues (11,35).

Each oligomer was simulated using the AMBER 12 (36) program suite (with the *pmemd* module for GPUs) (37), using our well-established multistep protocol (35,38) which involves energy minimizations of the solvent, slow thermalization and a final re-equilibration for 10 ns, prior to the 0.5 μs production runs. All simulations were carried out in the isothermal-isobaric ensemble (T = 298 K, P = 1 atm) using the parm99 force field (39,40) with the bsc0 modification for DNA (41) and Dang *et al.* parameters for ions (42–44). Long-range electrostatic effects were treated using the Particle Mesh Ewald method (45) with standard defaults, using a real-space cutoff of 10 Å. The length of chemical bonds involving hydrogen were restrained using SHAKE (46) and the Berendsen algorithm (47) was used to control the temperature and the pressure, with a coupling constant of 5 ps. Center of mass motion was removed every ps to limit the translational kinetic energy of the solute.

#### Complementary non-standard MD simulations

To highlight the role of the cations in the twist polymorphism, and their coupling with the backbone transitions, we performed three complementary 0.5 μs MD simulations starting from the equilibrated oligomer CGCGTCGACGCG. These simulations were performed with the previously described force field and protocol, but differed in the following ways:

- Langevin dynamics: One simulation was performed within the Generalized Born implicit solvent approximation (48,49) to check for average ionic strength effects (without explicit ions) on CG polymorphism. During the simulation, non-bonded interactions were calculated with a cutoff of 18 Å, and the salt concentration was set to 0.15 M. Temperature was controlled using a Langevin thermostat (50,51) with a friction constant of 5 ps^−1^ to mimic pure water viscosity. The random seed generator of the stochastic force was randomly changed at every simulation restart (every 50 ns) to avoid quasi-periodic oscillations (52).
- Heavy cations: One simulation was performed in explicit solvent using potassium chloride but increasing the mass of the potassium cation by a factor of 10^3^, while chloride was left unchanged. This allowed us to analyze the role of cation dynamics in CG polymorphism. These model calculations, which are very useful for qualitatively testing the relationship between cations and twist movements, should be treated with caution since the system is forced into physically unrealistic conditions.
- Adenines without H8 (H8^(-)^): One ‘prove of concept’ simulation in explicit solvent with sodium chloride was carried out removing the H8 atom of the two adenines flanking the CG step (one in the Watson and the other on the Crick strand). To maintain the total charge of the system, in these model calculations the H8 charge was transferred to the C8 atom. This simulation was aimed at understanding the role of H8 interactions in CG polymorphism.

#### Potential of mean force calculations

**Figure 1.**
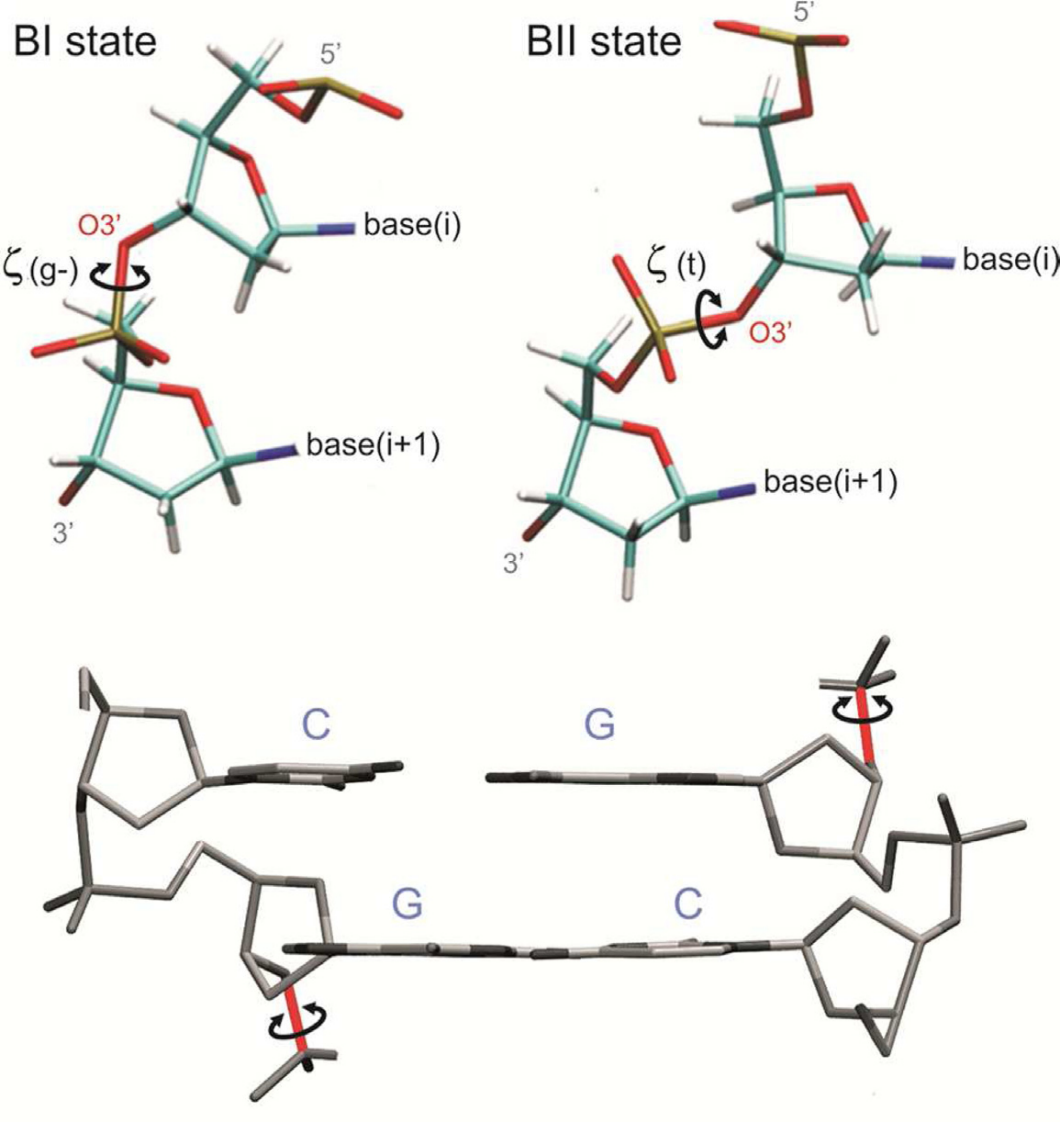
Representation of the ζ states under the BI or BII conformations. Unless otherwise stated, during this work we consider the coupling between the twist at the CG step and the two ζ angles (one in each strand) located at the 3′-junction of the step (highlighted in red in the bottom representation). Considering two ζ angles gives four possible combinations: (i) Both strands are in g-/g-, (ii) the Watson strand is in g- and the Crick strand in t (g-/t), (iii) the inverse situation (t,g-), and (iv) both strands are in t/t.

We calculated the relative free energy of the transition associated to the torsional change of the zeta (ζ) angles for the two flanking 3′-junctions of the CG step of the 18-mer sequence named GAAC by the ABC consortium (18) (this oligomer contains three copies of the ACGA tetranucleotide). For this purpose, we constructed the potential of mean force (PMF) (53,54) of the g-/g- to t/t transition (passing through the intermediate states g-/t and t/g-), using a harmonic biasing potential with a force constant value of 0.02 kcal mol^−1^ deg^2^ (see Figure 1 for the definition of the ζ angles chosen). We performed four separated one-dimensional calculations (transitions numbered from 1 to 4 in Figure 9): (i) from g-g- to g-t, (ii) from g-t to tt, (iii) from g-g- to tg- and (vi) from tg- to tt. In our nomenclature, the first substate refers to the ζ angle at the 3′ side of the CG step in the Watson strand and the second substate to the same angle in the Crick strand (as shown in Figure 1). The PMFs were always carried out from the canonical g- substate (characterized by an average ζ value of 270°) to the t substate (ζ ∼ 360°), one strand at a time. For example, when going from g-g- to g-t, the ζ angle in the Watson strand was fixed at 270°, and the ζ angle in the Crick strand was changed from 270° to 360° using restrained windows every 10°. Starting configurations for PMF simulations were extracted from the last snapshot of a 300 ns production run, simulated according to the ABC protocol (18). After sampling each window around the corresponding ζ value during 2 ns, biased probability histograms were obtained and weighted using the WHAM method (54).

**Figure 2.**
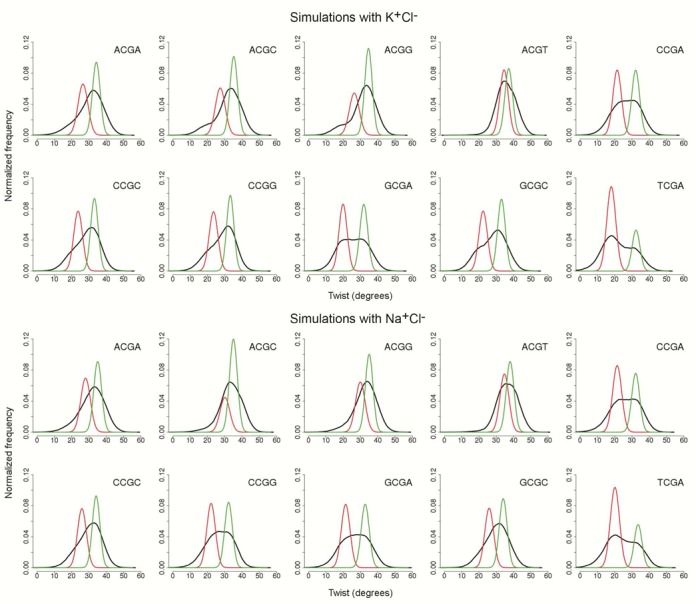
Twist distributions for the central CG step (black) and normal components obtained with BIC (LT component in red, HT component in green) for the 10 possible tetranucleotides.

**Figure 3.**
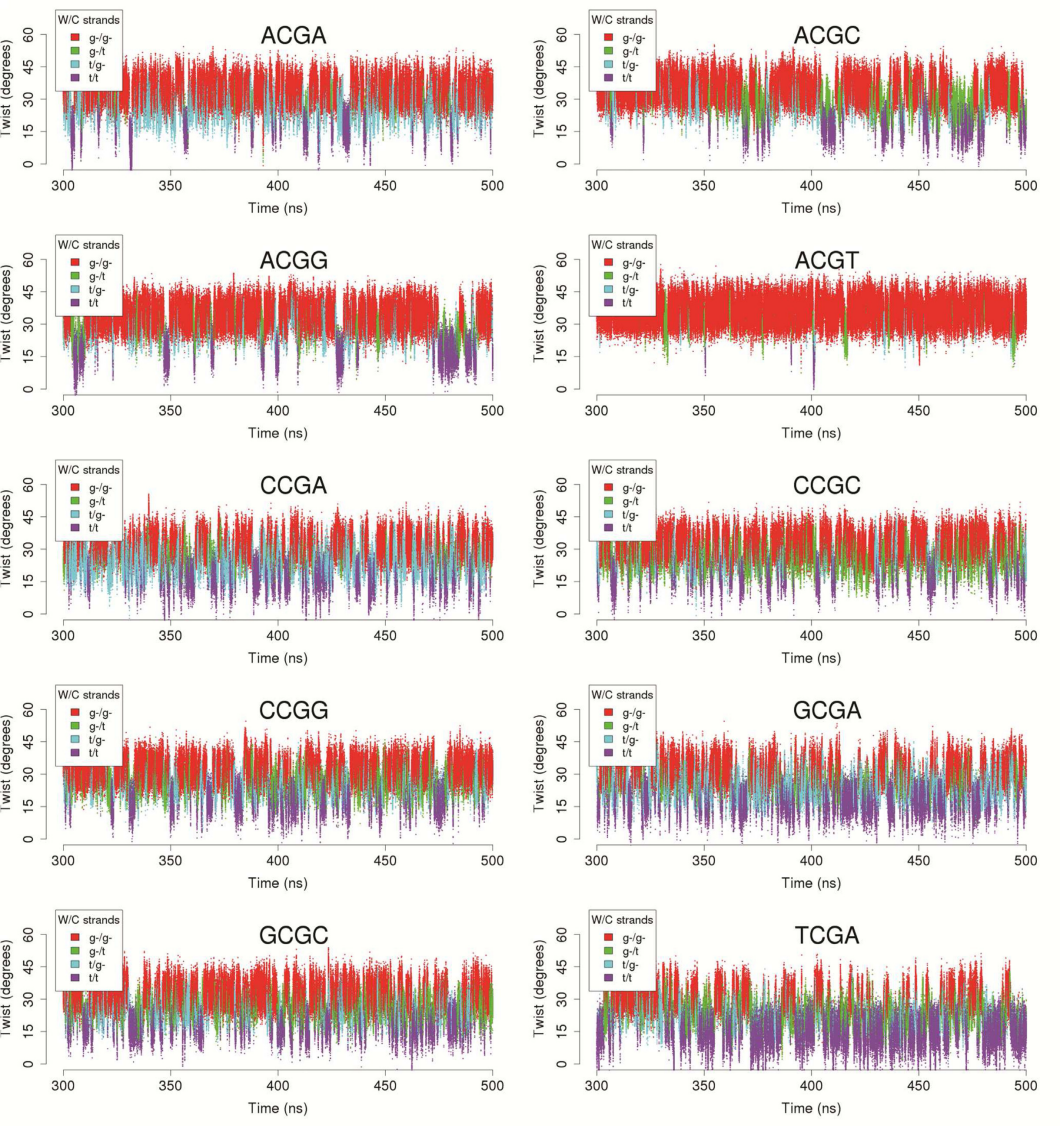
Correlations between twist at the central CG step and the states of the ζ angle at the 3′-side for K^+^Cl^−^.

**Figure 4.**
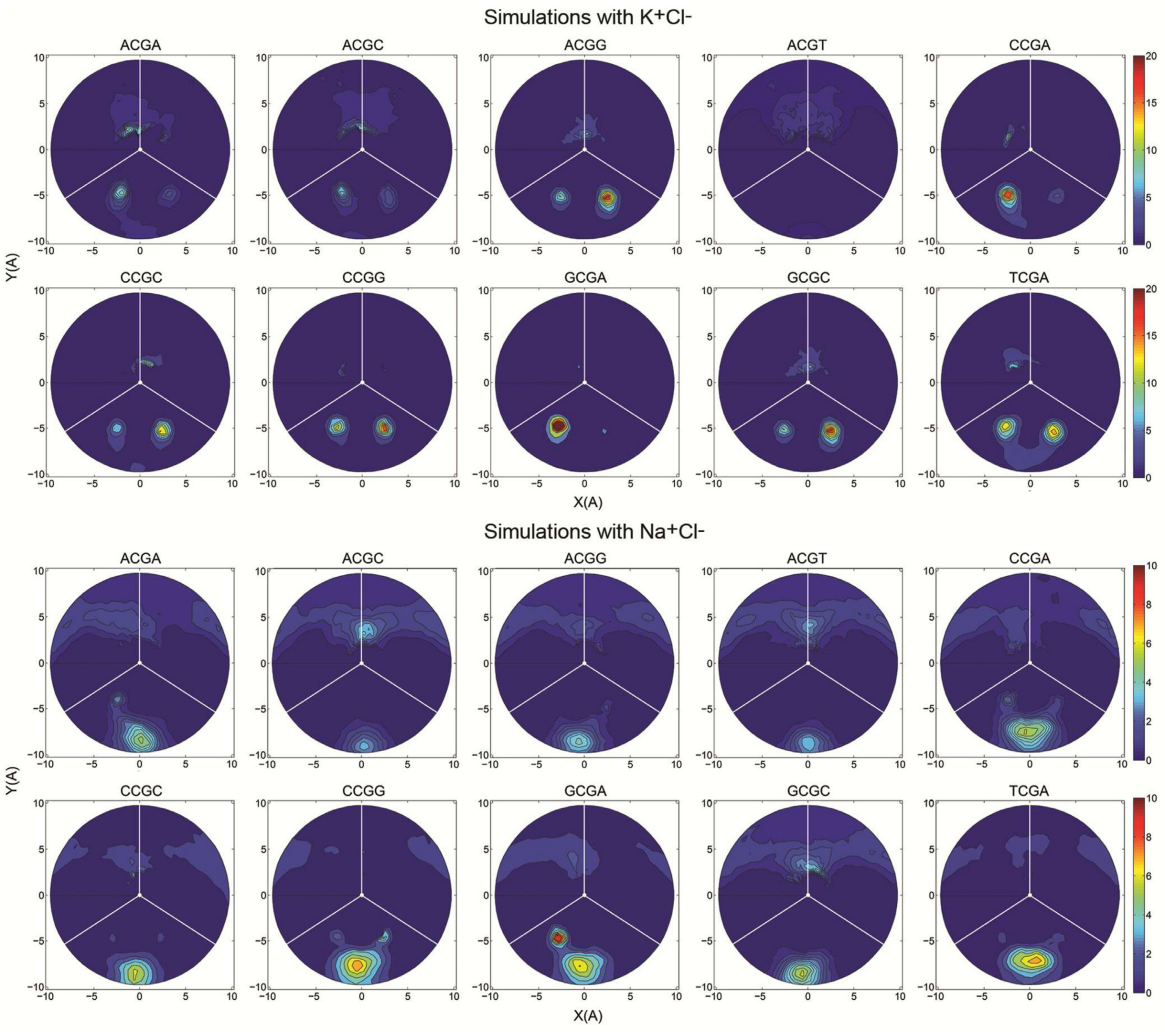
Two-dimensional cation distributions averaged over the last 200 ns of the trajectories. The plot show the radial-angular plane at the central CG step, the minor groove limits as white lines and the center of the major groove as a vertical radial vector. The results are plotted as molarities as shown by the color bars, with a blue to red concentration scale that goes from 0 to 20 molar for K^+^ and 0 to 10 molar for Na^+^.

**Figure 5.**
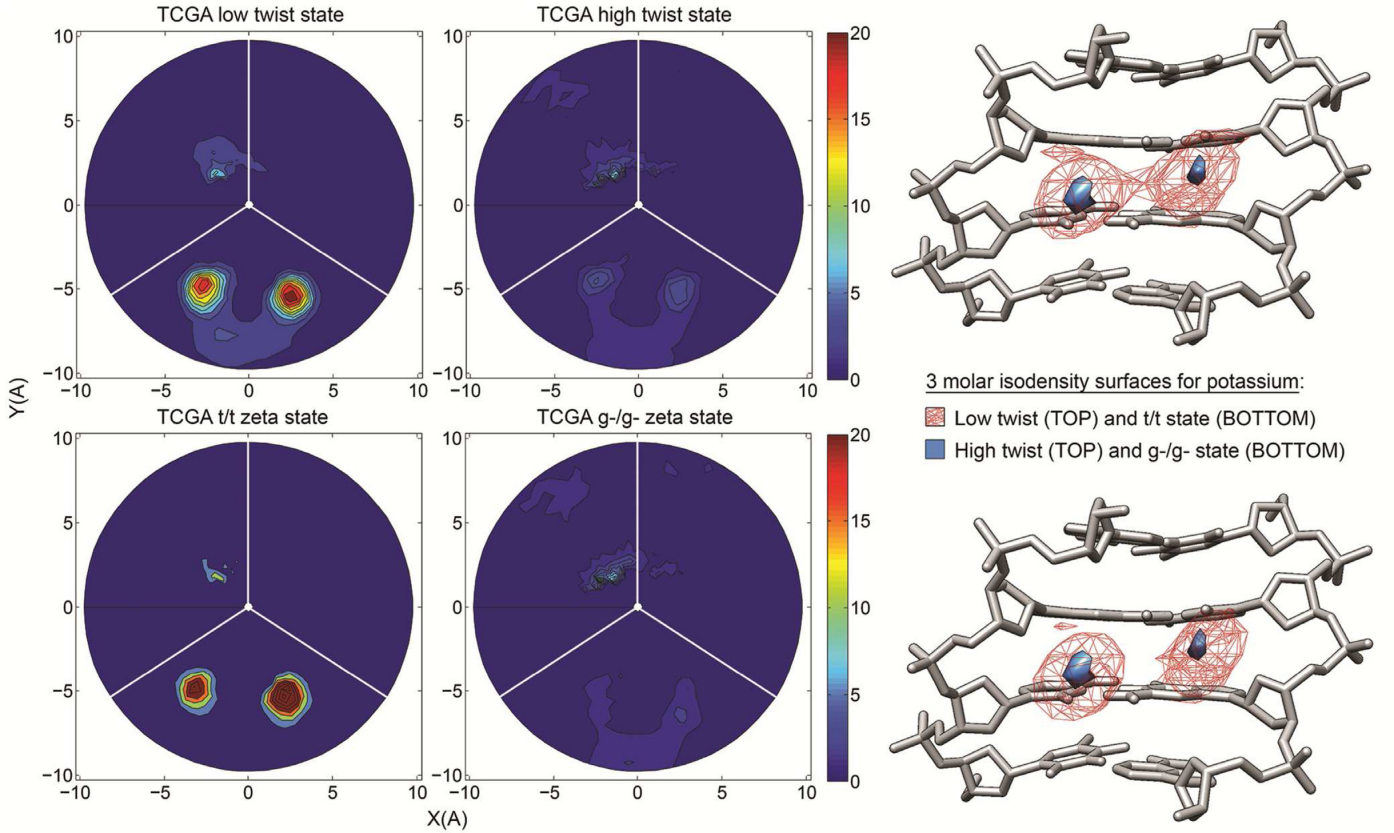
Two-dimensional K^+^ distributions obtained by filtering the TCGA trajectory according to either the twist (top panel) or the ζ (bottom panel) states of the CG step. The plots on the left show the radial-angular plane at the central CG step, the minor groove limits as white lines and the center of the major groove as a vertical radial vector. The results are plotted as molarities as shown by the color bars, with a blue to red concentration scale that goes from 0 to 20 molar.For sake of comparison, on the right, the three-dimensional distribution plots display the same molarity isodensity surface of 3 molar.

**Figure 6.**
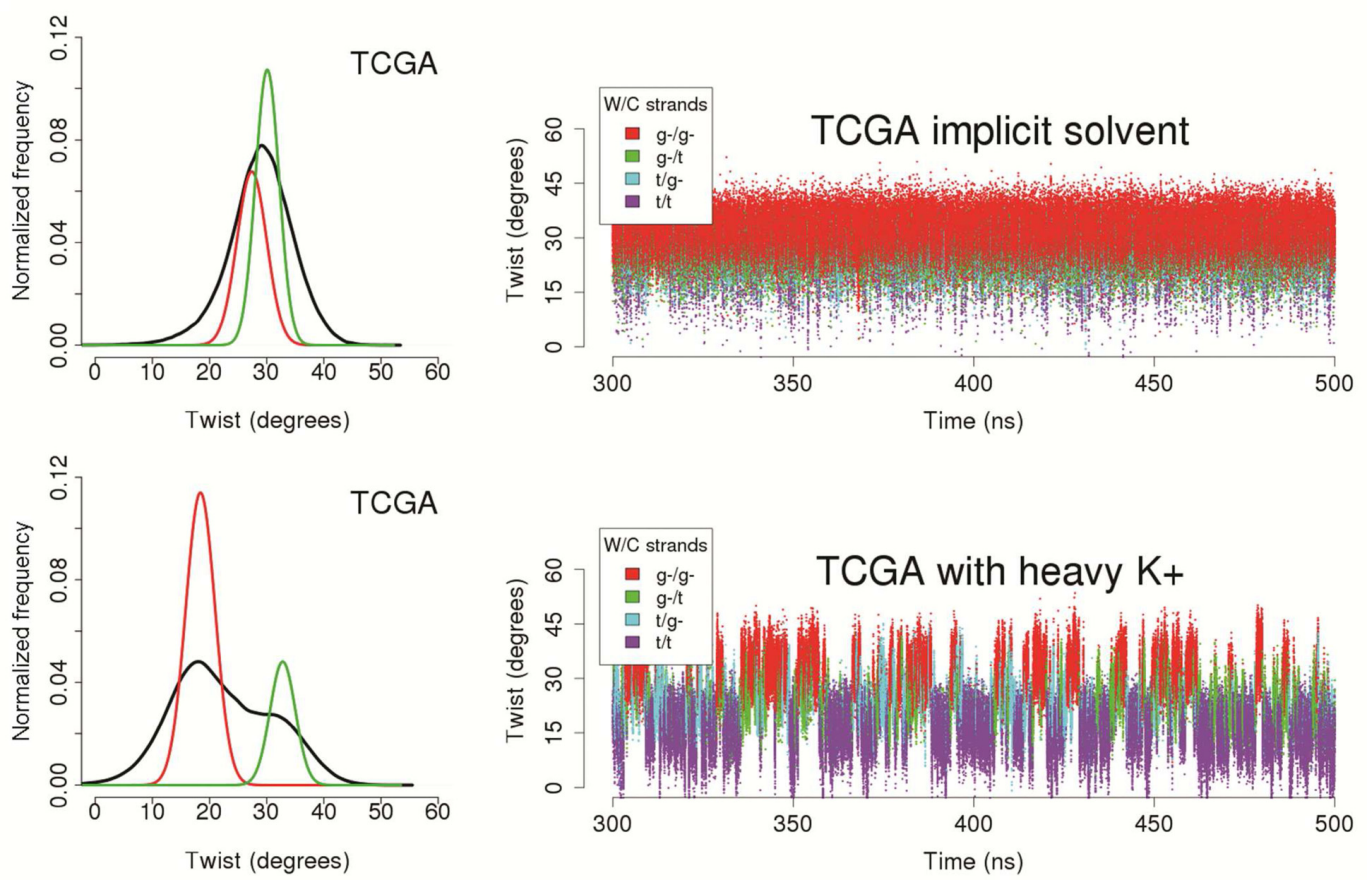
Twist distribution and correlation between twist and the possible states of the ζ angles. On the left, the observed distribution is depicted in black and normal components obtained with BIC in red (LT component), and in green (HT component) respectively. Correlation between the twist of the central CG step and the four states of the ζ angle at the 3′-junction are shown at the right of the distributions.

**Figure 7.**
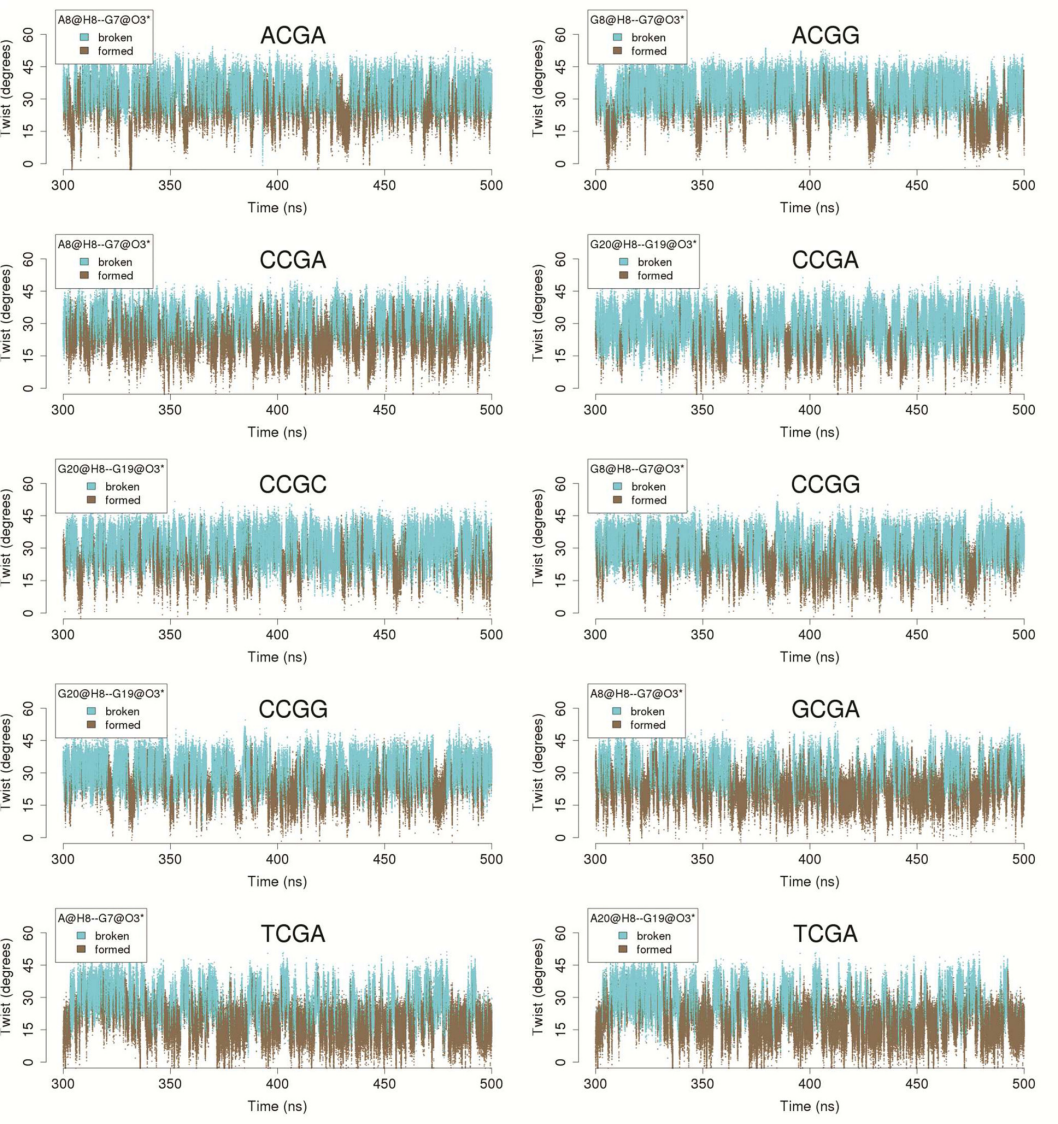
Time evolution of the twist at the CG step and the formation of the intra-molecular CH···O interaction. Results for the 10 possible tetranucleotides simulated in K^+^Cl^−^.

**Figure 8.**
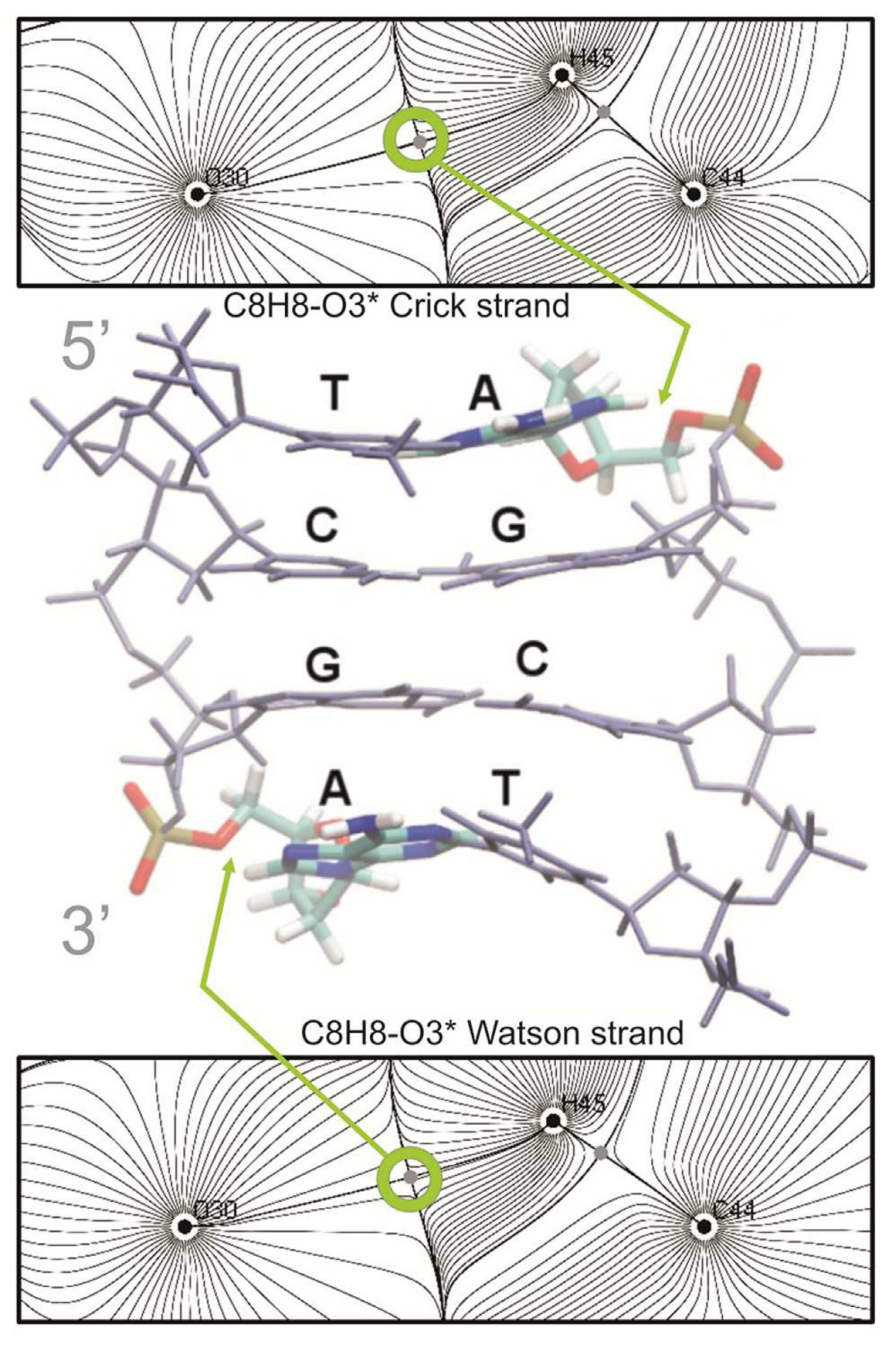
Hydrogen bond AIM analysis for the TCGA tetranucleotide in the BII/BII conformation. The atoms labeled as C44, H45 and O30 represent the C8, H8 and O3′ atoms of the flanking purine. The bond critical points are evidenced by gray dots. The nuclear critical points (located at the position of the nuclei) are shown by black dots, while the basin paths and the gradient field are depicted by gray lines. The bond paths, defined by the chosen two-dimensional projection (plane), are shown by black lines.

**Figure 9.**
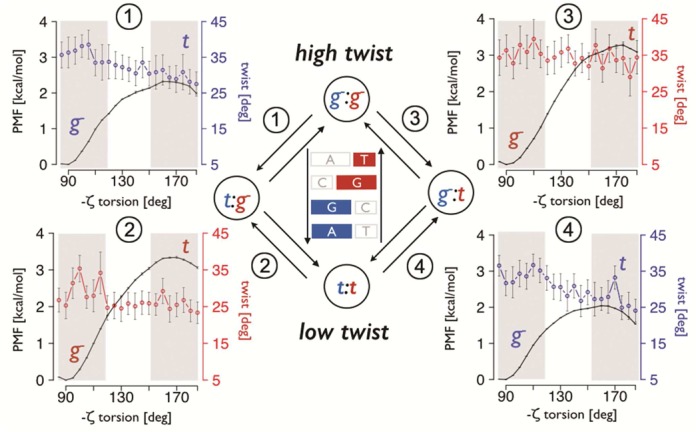
PMF simulations performed with sodium on the ACGA tetranucleotide. All the possible ζ transitions from g-g- to tt that go through an intermediate state were considered.

### Analysis of the trajectories

During production runs, data were collected every 1 ps, leading to more than 2 × 10^7^ structures. To ensure the convergence of the twist oscillations (11), and the convergence of the cation atmosphere around the DNA (55), only the last 200 ns of each simulation were used to produce the results (see Supplementary Figure S1 in supplementary material). All the trajectories were pre-processed with the *cpptraj* module of the AmberTools 13 package (36), DNALIVE (56) and local tools developed in the group (http://mmb.pcb.ub.es/www/tools). DNA helical parameters and backbone torsion angles associated with the CG step and its neighboring steps were measured with the Curves+ and Canal programs (57). To analyze the effect of the solvent, the last 50 ns of each trajectory were kept and all water molecules were analyzed.

#### Analysis of the cations with the Canion tool

**Figure 10.**
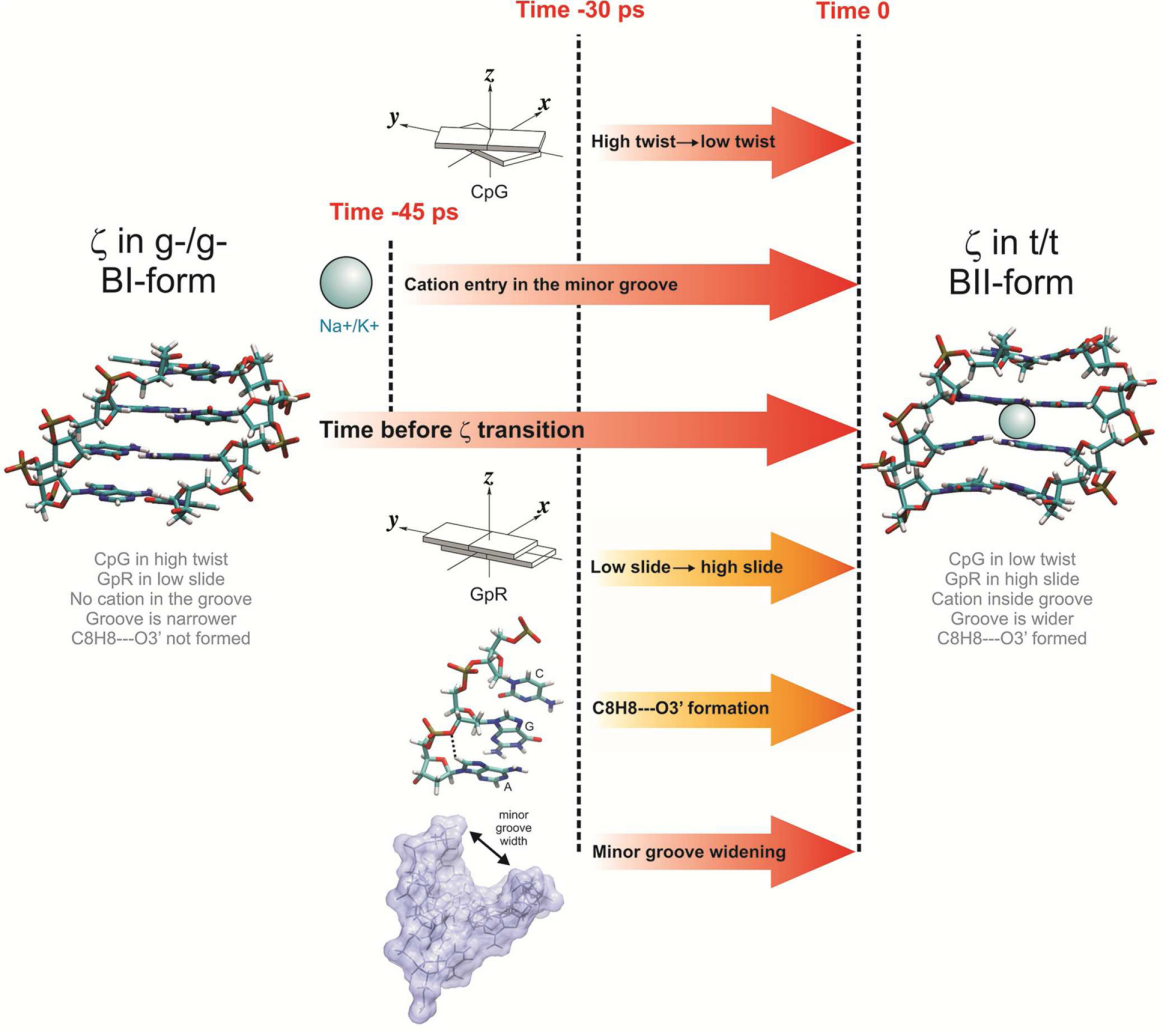
Schematic timeline of the concerted events that start with the entering of the cations in the minor grooves of the CG steps. The events are measured before a ζ transition when going from g-/g- to t/t. The arrows in red correspond to events that occur in all the tetranucleotides. Yellow arrows correspond to events that only occur when a purine is flanking the CG step at the 3′ side.

The new module from Curves+ (55) was used to determine the position of each cation in curvilinear cylindrical coordinates for each snapshot of the simulations with respect to the instantaneous helical axis. Given a distance *D* along the helical axis, ion distributions were computed for the CG step (defined here as N-0.2 ≤ *D* ≤ N+1.2 for a generic base pair step NpN+1) inside the grooves (*R* ≤ 10.25 Å), dividing the contribution between the minor groove (*A* = 33º to 147º) and the major groove (*A* = 33° to 0° to 147°) (55). We analyzed the ion distribution in 1- (*R*, *D*, *A*) and two-dimensional (*RA*, *DA*, *DR*) curvilinear cylindrical coordinates. Note that in the case of a two-dimensional radial-angular (*RA*) analysis, we used polar coordinate plots to make the results easier to understand. Three-dimensional distributions were also constructed in Cartesian coordinates using an average structure for the DNA oligomers obtained from the simulations with *cpptraj*. Ion densities were obtained in units of molarity as detailed elsewhere (55). To construct the choreography of events depicted in Figure 10, twist results were obtained by counting ions in the minor groove of the CG step 250 ps before each transition to CG low-twist and then averaging over thousands of such transitions to obtain ion populations as a function of time to ζ transition. This procedure was carried out using a utility program that reads the ion counts (produced by *Canion* following the Curves+ analysis) and twist values (produced by *Canal*) for each snapshot of a chosen trajectory. In the same way other parameters computed with *Canal* (or the *cpptraj* utility from AmberTools) could be traced (i.e. minor groove width, slide polymorphism, C-H···O hydrogen bond formation, etc). We computed the ratio between successful/unsuccessful K^+^-bonding events that lead to HT→LT transitions by inspecting an extended trajectory (1 μs -1 000 000 structures-) of the same oligomer used in the PMF calculations. We looked at the 3′-ζ of the CG step in the central ACGA tetranucleotide (C8pG9). We considered ‘states’ with or without ions that last at least 40 ps and then calculated the probability of BII/BII per ps during the last 20 ps of each ‘state’. The ratio of these results gives the influence of the ions. We divided the ion density into inner (R < 10.25 Å) and outer (10.25 Å < R < 15.0 Å) regions, into minor and major grooves and into steps 7–8, 8–9 and 9–10. The same procedure was followed to analyze the influence of Na^+^ around the C3pG4 step using 2 μs of simulation (2 000 000 structures).

#### Classical molecular interaction potentials

Our classical molecular interaction potential (cMIP, (58)) was used to analyze the ability of DNA to recognize sodium. The electrostatic interaction term was determined by solving the linear Poisson–Boltzmann equation (59), while the van der Waals contribution was determined using standard AMBER Lennard–Jones parameters (40,58). The ionic strength and the reaction-field dielectric constant were set to 0.15 and 78.4 M, respectively, while the dielectric constant for DNA was set to 8 (60). The calculations were performed using the same average structure used to extract the helical axis for the Cartesian coordinate cation analysis previously described.

### Quantum mechanical calculations

To make a first principles confirmation of the existence of the CH···O intra-molecular ‘hydrogen bond’, Bader's atoms in molecules (AIM) electron topology analysis was performed (61–63). Five representative snapshots from the three selected tetranucleotides (CCGA, CCGG and TCGA) in the BII/BII-LT conformation, were extracted from the MD simulations to perform single-point MP2 calculations. Waters and ions were removed, and only the adjacent 5′-GpA-3′ or 5′-GpG-3′ dinucleotide was kept and subjected to single-point calculations at the MP2(FC)/6–31G(d,p) level of theory using Gaussian 09 (64). H atoms were used to complete the valence of the 5′ and 3′ oxygen atoms. AIM analysis provides evidences for bond critical points (bcp) between the C8H8(A/G)···O3′(G) or C8H8(A/G)···O5′(G) hydrogen bonds. The electron density (ρ), the gradient (▽ρ) and its Laplacian (▽^2^ρ) were computed to measure the strength of the interaction. AIM analysis was performed with AIM-UC (65) for plotting density and gradient paths, while the value of the electronic density and its derivative at the bcp were calculated with the AIMQB module within the AIMAll package (66).

### Database analysis

Database analysis was carried out at both molecular and genomic levels. At the molecular level, we analyzed a set of crystal structures of DNA with small, intercalated molecules. At the genomic level, we computed the frequency of bi, tri and tetranucleotides containing the CG step in the genomes of four model organisms.

#### X-ray structures of DNA with small intercalators

74 high resolution (<2.5 Å) X-ray crystal structures of DNA oligomers with small intercalated molecules were retrieved from the Protein Data Bank (67). We paid special attention to the sequence context at the intercalation site and to the values of the helical parameters computed with Curves+ (57). The PDB ID of the complexes studied are: 110d, 151d, 152d, 182d, 198d, 1agl, 1c9z, 1d10, 1d11, 1d12, 1d14, 1d15, 1d17, 1d21, 1d22, 1d32, 1d33, 1d35, 1d36, 1d37, 1d38, 1d54, 1d58, 1d67, 1da0, 1da9, 1dl8, 1eg6, 1fdj, 1fdg, 1fn1, 1fn2, 1imr, 1ims, 1jo2, 1k9g, 1kci, 1l0r, 1m69, 1n37, 1nab, 1p20, 1qch, 1r68, 1rqy, 1xc5, 1xcq, 1z3f, 215d, 224d, 234d, 235d, 236d, 245d, 258d, 276d, 277d, 278d, 288d, 2d34, 2des, 2gb9, 2gjb, 2gwa, 308d, 366d, 367d, 380d, 385d, 386d, 3ft6, 452d, 465d, 482d. In 74% of the complexes (49 structures), the intercalation occurs at a CG step. It is worth noting that in almost all the cases, the CG steps with the intercalators are located at the first and/or the last position of the DNA oligonucleotide, and hence are subjected to strong end and packing effects, which may affect the local helical conformations. A second source of bias of the dataset could be due to the preferences of experimentalists to favor sequences that have previously been successfully crystalized.

#### Genomic oligonucleotide frequency

The di-, tri- and tetranucleotide composition analyses of genomes were carried out using R/Bioconductor (68). *Homo sapiens* (UCSC version hg19), *Saccharomyces cerevisiae* (UCSC version SacCer3), *Caenorhabditis elegans* (UCSC version ce10) and *Drosophila melanogaster* (UCSC version dm3) genomes were studied. We considered all the overlapping di-, tri- and tetranucleotide sequence fragments containing the CG step for the complete genomes. The frequencies of complementary segments were summed. For the sake of comparison, the occurrences were normalized by the length of the genomes (when comparing different organisms), or additionally by the length of each region (when looking to the CG occurrences in introns, exons, promoter regions, etc).

### Statistics, graphics and molecular plots

The statistical analysis, including the Bayesian Information Criterion (BIC), linear correlations and all the plots were performed with the R 3.0.1 statistical package (69). The two-dimensional *RA* molarity plots were obtained with Matlab 6.2 (70) using scripts available on the Curves+ web page (http://bisi.ibcp.fr/tools/curves_plus/matlab-scripts.html). The molecular plots were generated using either VMD 1.9 (71) or the UCSF Chimera package version 1.8.1 (72).

## RESULTS AND DISCUSSION

### Statistical analysis of the twist distribution and correlation between twist and the ζ torsion angles

The Bayesian Information Criterion (BIC) (73) was used as previously reported (11), limiting the analysis to only two components to determine the number of normal functions needed to meaningfully represent the appearance of possible substates in the twist distributions. As expected from our previous study at the dinucleotide level (11), the twist distributions at CG steps should be described not by an average value and an associated standard deviation, but by using two averages, two associated standard deviations and a mixture proportion. The average values, the standard deviations and mixture proportions obtained using BIC for the 10 possible tetranucleotides are presented in Table 1, and twist profiles are shown in Figure 2. Note that while the results obtained for Na^+^ and K^+^ counterions are very similar, the tetranucleotide environment has a very large impact on the global twist distribution at the CG step in different ways: (i) the weight of the LT and HT distributions change (from 0.7/0.3 (LT/HT) for TCGA to 0.32/0.68 (LT/HT) for ACGA), (ii) the average twist values of LT and HT states change quite dramatically and finally (iii) in some extreme cases (e.g. ACGT and ACGC) the HT and LT values are so close that the distribution can be clearly considered unimodal, since HT↔LT transitions are very infrequent (see Figure 3) and the twist distribution can be reasonably represented by a single Gaussian (see Figure 2).

**Table 1.** Twist weighted averages, and BIC components for the 10 possible tetranucleotides simulated with K^+^Cl^−^ and Na^+^Cl^−^

	1^st^ component			2^nd^ component			Weighted average
	Avg	Std. dev.	Weight	Avg	Std. dev.	Weight	
K^+^Cl^−^							
ACGA	26.5	8.2	0.47	34.2	5.0	0.53	30.6
ACGC	27.4	8.2	0.44	35.2	4.9	0.56	31.8
ACGG	26.4	8.3	0.39	34.6	4.7	0.61	31.4
ACGT	34.5	6.0	0.52	37.2	5.0	0.48	35.8
CCGA	21.4	6.1	0.52	32.1	5.2	0.48	26.5
CCGC	23.7	6.5	0.49	33.1	4.7	0.51	28.5
CCGG	23.5	6.5	0.49	33.2	4.4	0.51	28.5
GCGA	19.9	5.5	0.51	31.9	5.3	0.49	25.8
GCGC	22.4	6.2	0.48	33.0	5.0	0.52	27.9
TCGA	18.0	6.7	0.70	32.3	5.0	0.30	22.2
xCGy	24.4	6.8	0.50	33.7	4.9	0.50	28.9
Na^+^Cl^−^							
ACGA	28.0	7.6	0.48	35.0	5.2	0.52	31.6
ACGC	30.1	7.9	0.32	35.0	5.2	0.68	33.5
ACGG	29.9	7.7	0.45	35.0	4.8	0.55	32.7
ACGT	34.7	6.7	0.49	38.0	5.1	0.51	36.4
CCGA	21.4	6.9	0.56	32.1	5.3	0.44	26.1
CCGC	25.9	6.6	0.49	34.1	4.8	0.51	30.1
CCGG	22.1	6.3	0.52	32.2	5.1	0.48	26.9
GCGA	21.4	6.4	0.52	32.7	5.5	0.48	26.8
GCGC	25.9	6.5	0.49	34.0	5.2	0.51	30.0
TCGA	20.1	7.0	0.69	33.5	5.0	0.31	24.3
xCGy	26.0	7.0	0.50	34.2	5.1	0.50	29.8
GCGA^a^	25.4	7.3	0.48	37.9	4.6	0.52	31.9

^a^Computed from the 4 μs long simulation of the Drew–Dickerson dodecamer (bps C3pG4).

As previously suggested (18), the twist polymorphism at CG steps is correlated with conformational transitions for ζ states (Figure 3 and Supplementary Figure S2, for K^+^ and Na^+^ respectively) at the 3′-side of the CG step (see Figure 1). Rotations around ζ (coupled to changes in the adjacent ϵ dihedral) define the so-called BI and BII states of DNA (g- and t conformers for ζ lead respectively the BI and BII state of each phosphodiester junction, see Figure 1). The HT population is found mostly with the g-/g- conformation (BI/BI), while, for the LT population, the ζ angles are almost exclusively in the t/t (BII/BII). This coupling is clearly seen in the integrated population for sequences, e.g. TCGA, that exhibit a large population of LT and have a low BI/BII ratio (45/55; averaging Na^+^Cl^−^ and K^+^Cl^−^ results) (see Figure 2). However, coupling between LT/HT is visible even for the steps such as ACGT, where the population of LT population is small and where, as expected, the BI/BII ratio is large (96/4). We can conclude that BI/BII polymorphism is an intrinsic property of CG steps and is related to the HT/LT states, as suggested by both experimental and theoretical studies of ‘base destacking’ (18,74,75). In passing, note that the sequence-dependent trend found for the propensity of BII states obtained from these simulations is in excellent agreement with earlier NMR experiments (76).

Due to the inherent difficulty in unambiguously separating the HT and LT states from the twist distributions (even using the BIC components), we used the well-defined states of the ζ angles at the 3′-side of the CG step to filter the trajectories and obtain several of the observables discussed in the next sections.

### Interaction of cations with the minor groove of CG steps: correlations with twist, BI/BII states, minor groove width and flexibility

We have followed the trajectories of each individual cation during the simulations to determine cation occupancies and residence times (to avoid equilibration problems only the last 200 ns of trajectories were used here). As shown in the radial-angular density maps (Figure 4), cations tend to concentrate in the minor groove of CG steps, with a significant sequence-dependence: high concentration in the cases with largest proportion of LT state (e.g. TCGA, GCGA or CCGA) and low concentration for the less polymorphic tetranucleotide (e.g. ACGT and ACGC). Na^+^ and K^+^ show, roughly, a similar ability to occupy the minor groove (see Table 2) of the CG step, although the Na^+^ concentrations are always lower respect to K^+^. For the large K^+^ cation, two minor groove regions are equally (but not simultaneously) populated (Figure 4), while for the smaller Na^+^ there is only one preferred region. Despite having a larger ionic radius K^+^ cations can penetrate deeper inside the groove, which could be related to the fact that potassium is more easily desolvated and thus can interact more directly with DNA. Indeed, in agreement with the density maps (Figure 4), when the cations are inside the minor groove of the CG step, Na^+^ has a higher average number of water molecules in the first coordination shell than K^+^ (4.8 versus 3.4 waters per cation respectively). Average ion residence times inside the minor groove are quite low, typically below 10 ps (see Table 2), confirming previous claims on the high cation mobility within the grooves (35,77). The longest groove residence times can reach almost 1 ns, while states without any cation in the groove can exist for more than 2 ns. The presence of one cation in the CG minor groove largely hinders the entrance of a second one, since in 98.500% of the time when a cation is in the groove, it is found partially solvated and alone. Only during 1.499% of the time the presence of two cations can be detected, and three ions are simultaneously in the same area of the minor groove only 0.001% of the time.

**Table 2.** Potassium and sodium dynamics in the minor groove of CG step

	ACGA	ACGC	ACGG	ACGT	CCGA	CCGC	CCGG	GCGA	GCGC	TCGA
K^+^Cl^−^										
Average residence time (ps)	6.0	5.0	5.6	3.6	7.3	6.7	5.7	7.9	6.9	11.3
Occupancy (%)	12.8	13.6	15.2	4.5	19.6	17.8	17.6	26.0	18.7	30.8
Transitions (count)^a^	4271	5419	5468	2476	5373	5316	6182	6618	5413	5446
Na^+^Cl^−^										
Average residence time (ps)	6.7	5.6	6.2	7.1	6.1	5.4	5.7	6.5	6.9	5.8
Occupancy (%)	18.1	9.2	14.5	12.1	21.7	18.4	25.6	23.8	19.5	24.0
Transitions (count)	5425	3308	4653	3409	7144	6777	9012	7307	5664	8267

^a^Over a total of 200 000 structures analyzed (the last 200 ns of each trajectory).

Analysis of the most polymorphic tetranucleotides demonstrates that the LT state (ζ t/t, i.e. BII/BII) concentrates more cations in the minor groove that the HT state (see e.g. d(TpCpGpA) in Figure 5 and Supplementary Figure S3 for K^+^ and Na^+^ respectively). This can be partially, but probably not totally, explained by simple electrostatic considerations (see cMIP maps in Supplementary Figures S4 and S5).

We conclude that LT/HT polymorphism correlates not only with BI/BII transitions, but also with the cation density in the minor groove. Additional parameters such as the minor groove width or the stiffness of the CG step, that should be relevant in the interaction between DNA and its environment (35,78), were correlated with the cation occupancy and the twist polymorphism. The results shown in Supplementary Figure S6, are indicative of a strong correlation between all these factors. Accordingly, we have a complex choreography of coordinated changes involving apparently disconnected descriptors (see Supplementary Tables S1 and S2 and Supplementary Figure S6). Thus, cation density and the weighted average twist correlate with R^2^ = 0.9 (average for Na^+^ and K^+^); the weighted averaged twist and the BII percentage correlate with R^2^ = 0.99; and, not surprisingly, cation density and BI/BII ratio correlate with R^2^ = 0.9 (See Supplementary Table S2). In summary, the LT state implies a sharp increase in the BII population, an enrichment of cations in the minor groove of CG steps, a decrease in the apparent stiffness of CG steps and an increase in the width of the minor groove. All these coordinated changes are logical from a mechanical point of view, except perhaps the increase in cation density when the minor groove is wider. This finding is in contrast with previous experimental (79) and simulation (35) results obtained from the analysis of the AATT minor groove, and with the theory of ion condensation (80,81), that would suggest that the presence of the cation screening phosphate charges would lead to an easier approach of these groups. Two hypotheses, that would deserve more attention, could be relevant: (i) The rule of cation condensation described for AT-grooves differs in the case of CG steps, where ion attraction of phosphate groups cannot compensate for the increase in minor groove width required for DNA untwisting; and (ii) in particular for Na^+^, where the interaction with DNA is mainly mediated by water molecules, the existence of a ‘floor’ of waters within the minor groove could lead to a pronounced widening of the groove (82).

To highlight the relevance of cations in the polymorphic behavior of the CG steps, we performed two additional 0.5 μs MD simulations for the duplex containing the TCGA sequence using, in one case, implicit solvent (GB/SA implementation: where the cation screening was introduced macroscopically by the ionic strength, but not explicitly, see ‘Materials and Methods’ section) and, in the other case, explicit solvent and K^+^, but artificially increasing the cation mass by a factor of 10^3^ (which should significantly slow down the dynamics of the cation). As shown in Figure 6, despite the existence of some transient LT states coupled with the ζ torsion, no significant polymorphism is found in the absence of explicit cations, indicating that polymorphism cannot be explained by general ionic screening considerations, but requires specific cations placed at specific positions in the minor groove. With the introduction of the heavy potassium (Figure 6), the average residence time and the occupancy of the cations in the minor groove increased (from 11.3 to 15.7 ps, and from 31 to 40%, respectively). Longer and more sustained LT state can be seen and the LT population is slightly increased, maintaining the features of the BIC components (averages and associated standard deviations, see Table 1). These model calculations emphasize the key role exerted by specific cations for the twist polymorphism of CG steps.

### The importance of an unusual CH···O ‘hydrogen bond’ in the BII substate and the slide polymorphism of the flanking d(GpR) steps

Figures 2 and 3 show that the presence of a purine (R) on the 3′-side of the CG step (defining the next GR step) enhances CG polymorphism (with R = A > G). Conversely, the presence of a purine in the 5′-side significantly reduces the bimodal behavior of CG twist. Analysis of the collected ensembles highlighted the existence of an unexpected interaction between the C8H8 group of the 3′-flanking purine and the O3′ atom of the CG phosphodiester backbone (see Supplementary Figure S7). This interaction can lead to very close CH···O contacts, especially when adenine is in the 3′ position (Supplementary Figure S8). Interestingly (see Figure 7 and Supplementary Figure S9 for K^+^ and Na^+^ respectively), a clear correlation between the twist and the CH···O contact exists when the system is in a LT state, and accordingly the backbone is in the BII substate. The proton attached to the C8 has a weakly acidic nature (e.g. the charge of the H8 atom in adenine, derived with QM methods, is one order of magnitude more positive than the nearly chemically equivalent H2 proton (40)). The CH···O contact can consequently have a hydrogen bond nature (83,84). To confirm this hypothesis, we performed *ab initio Atoms in Molecule* (AIM) calculations (see ‘Materials and Methods’ section) for representative snapshots of the BII/BII substate for three selected tetranucleotides: CCGA, CCGG and TCGA. The chosen sequences have either two contacts (C8H8_(i+1)_···O3′_(i)_) between G and A (one GA step in each strand), two contacts between GG, or one of each type in the case of the CCGA sequence. As described in the 'Materials and Methods' section and explained in detail elsewhere (62), AIM analysis of the electron density can determine the stabilizing nature of any interaction. Results presented in Figure 8 (TCGA) and Supplementary Figure S10 (for CCGA and CCGG cases), clearly show bond critical points (bcp) between the H8 and the O3′ atoms in all cases, suggesting that this interaction is indeed stabilizing the system (85,86). The electron density associated to these bcp is larger than that found in CH···O interactions in nucleobase pairing (see Supplementary Table S3 and the reference values (87)), and in various aryl–Π interactions (85,86). In fact, the electron density at C8H8···O3′ bcp is not far below that of canonical hydrogen bonds (e.g. 0.025 a.u. for the N6H6···O4 hydrogen bond in canonical A-U pairing, or 0.028 a.u for the N3H3···O4 interaction in U-U pairing (87)). Considering the linear relationship between electron density at bcp (and the Laplacian) with the interaction energy, and using the reference values for A-U pairing (85,86), we can estimate that the stabilization provided by the C8H8···O3′ contact should be of the order of 3 kcal mol^−1^. This interaction is thus not very different from that of a canonical hydrogen bond. It can thus contribute significantly to maintaining the BII state and, consequently, the LT conformation. It is worth noting that water occupancy around the O3′ atom increases from 15% in the BII (LT) state to 37% in BI (HT), suggesting that the formation of the CH···O interaction could be partially compensated in the BI state by the formation of hydrogen bonds with explicit water molecules.

Finally, to verify the impact of the C8H8···O3′ interactions in the ensembles derived from MD, we performed an additional 0.5 μs MD simulation in explicit solvent for the DNA duplex containing the TCGA tetranucleotide. In this simulation, labeled H8^(-)^, we modified the force-field to remove the H8 atom of the adenines flanking the CG step, transferring its charge to the adjacent C8 atom (see ‘Materials and Methods’ section). The resulting changes are rather small, but are sufficient to strongly reduce the BII conformation and the LT state of the CG step (Supplementary Figure S11). As a result of this modification, the weighted average twist changes from 22.2°C in the normal calculations to 27.9°C in the H8^(-)^ calculations (Supplementary Figure S10), with cation occupancy moving from 24 to 21%. Clearly, this inter-residue C8H8···O3′ interaction has a major effect in modulating the HT-LT equilibrium and all the coupled changes, including cation entrance and the shift to the BII substate.

The surprising impact of C8H···O3′ contacts in CGR twist bimodality (R being purine), strongly suggests that it might be also behind the slide bimodality found by the ABC consortium (18) and in later MD studies (11) for RR steps. Analysis of the trajectories (see Supplementary Figure S12) clearly confirm the coupling of slide polymorphism and the formation of the C8H8···O3′ interactions (especially strong for adenines), with all the conformational changes described so far for the CG step. We have thus revealed an unexpected and complex choreography of changes involving non-canonical hydrogen bonds, ion movements and backbone rotations that link two apparently disconnected sequence-dependent polymorphisms.

### Thermodynamics of the backbone substates: unraveling the mechanism of backbone transitions

The last 200 ns, of the 0.5 μs simulations, were used to study the thermodynamics of the backbone substates. Since our trajectories were sampled every 1 ps, 200 000 structures were sorted according to their ζ state (g-g-, tg-, g-t, tt) and a transition matrix was built. The free energy associated to the change from one backbone state to the other was computed using the relative population respect to the reference g-g- ζ state. The results for sodium and potassium, which have typical standard deviations of 5.3º (with standard errors of 0.012) for twist and 0.2 kcal mol^−1^ (with standard errors of 0.004) for the energies, are presented in Table 3 and Supplementary Figure S4 respectively (comparison with a very long simulation of the Dickerson dodecamer confirm that the results presented here are acceptably converged). We found that the direct transitions between g-g- and tt ζ states are very rare, and typically occur through intermediate states where only one of the two 3′-ζ angles changes at a given time. A detailed analysis of the trajectories (see Supplementary Table S5) reveals that the 3′-ζ torsion that flips first is always the one between GA or GG steps, since in those cases the ζ−flip is stabilized by the C8H8···O3′ interaction. For example, in the case of GCGA, the rotation around the ζ of the GA junction of the Watson strand is favored 0.4 kcal mol^−1^ respect to the rotation around ζ in the GC junction of the Crick strand. As shown above, C8H8···O3′ contacts are stronger when the donor is adenine rather than guanine, a fact that is also visible when analyzing the CCGA tetranucleotide. In this particular case, the ζ−flip can lead to the formation of the C8H8···O3′ interaction in both strands; in the GA step of the Watson strand and in the GG step in the Crick strand. As shown in Supplementary Table S5, the first flip occurs in the Watson strand favoring GA over the GG step. It is also remarked that, as noted above, 5′-purines disfavor the ζ transition, and 5′-adenine largely inhibits twist polymorphism.

**Table 3.** Thermodynamics of the ζ (bp_(i+1)_ in the 3′ direction) states and associated average twist for sodium

	State relative free energy (kcal mol^−1^)				Associated average twist (degrees)			
	g-g-	tg-	g-t	tt	g-g-	tg-	g-t	tt
ACGA	0.0	0.5	1.8	1.3	35.2	28.0	27.1	17.3
ACGC	0.0	1.1	1.7	1.9	35.2	28.5	28.4	16.6
ACGG	0.0	0.8	1.4	1.5	35.0	28.6	27.4	17.5
ACGT	0.0	1.7	2.0	2.5	37.1	31.4	28.3	16.8
CCGA	0.0	0.1	0.8	0.1	32.9	25.3	26.8	16.8
CCGC	0.0	0.8	0.7	0.9	34.0	28.0	26.8	18.8
CCGG	0.0	0.8	0.7	0.9	34.0	28.1	26.8	18.8
GCGA	0.0	0.2	1.0	0.3	33.7	26.2	26.5	17.3
GCGC	0.0	0.7	0.7	1.0	34.0	27.0	27.2	19.0
TCGA	0.0	0.3	0.3	−0.2	33.4	26.2	26.3	16.3
Drew–Dickerson dodecamer (4 μs)								
GCGA	0.0	0.3	1.2	0.7	37.5	30.6	28.1	18.5

Potentials of mean force (PMFs; see ‘Materials and Methods’ section) were carried out to unambiguously prove the coupling between the backbone and the twist transitions, and to qualitatively validate the free energy of transition obtained from the counting of the ζ states. Figure 9 presents the data obtained for the ACGA tetranucleotide. Based on the results in Table 3, the transition from g-g- to tt occurred in two steps, always passing through an intermediate state, changing only one ζ angle from g- to t at a time (see *'*Materials and Methods' section).

PMF calculations show that an intermediate tg- ζ state is thermodynamically preferred by a little more than 1 kcal mol^−1^, in good agreement with the estimate obtained by state counting in Table 3. Clearly, this preference reflects the stabilizing effect of the CH···O interaction, which, in the case of the ACGA tetranucleotide, only exists for the GA step in the Watson strand, consequently favoring a first transition in this strand. Furthermore, when flipping ζ from g-g- to tg-, we found a coupled reduction in the twist angle (from 35° to 28°, see Table 3) in complete agreement with the average twist obtained in the unbiased simulations. When the final tt ζ state is reached, the average twist is ∼23°, slightly higher than expected, probably due to convergence problems inherent to restrained PMF calculations. Although the relative energies are in very good agreement, the absolute values are slightly different. In the PMF analysis, the intermediate and the final states are always around 1.5 kcal mol^−1^ higher with respect to the ζ g-g- reference, compared to the results reported in Table 3. Knowing that the transition free energies increase slightly when simulating beyond the microsecond (due to sampling issues; see Table 3), and considering that the energies derived from PMF calculations are always slightly overestimated as a consequence of the use of restraints, we can assume that the real transition free energy should be something in-between the two values (i.e. for the g-g- to tg- transition the intermediate ζ state should be unfavorable by 0.5 to 2.1 kcal mol^−1^; and, by analogy, going from g-g- to g-t is unfavorable by 1.8 to 3.2 kcal mol^−1^). This means that the presence of the CH···O interaction (that was observed in the small windows sampled during the PMF calculation), stabilizes the ζ transition on average by nearly 1.2 kcal mol^−1^.

### The relevance of the DNA-cation interaction: causality

The analysis of the temporal correlation between the twist states, the penetration of cations in the minor groove, the slide polymorphism in the next-neighbor GR steps, the formation of the CH···O interactions, the widening of the minor groove and the transitions between the ζ torsions, helped us to decipher the causality of the observed relationships. For this purpose, we used the 4 μs MD simulation of the Dickerson dodecamer (that contains a GCGA tetranucleotide) in order to obtain good statistics for the transitions occurring between all these components. From the 2 million structures analyzed (last 2 μs sampled every 1 ps) we could observe ∼100 000 LT↔HT transitions, ∼50 000 cation bound/unbound states in the minor groove and ∼25 000 ζ transitions. We developed an appropriate analysis code to trace the ions, the twist, the CH···O contact and the groove size, 250 ps before any ζ transition from g-g- to the final tt state (see Supplementary Figure S13). As shown in Supplementary Figure S14, we also investigated the reverse situation, namely recovering the canonical g-g- BI/BI conformation starting from ζ tt (BII/BII).

Combining all the components of this concerted choreography, we were able to build a timeline that gives an average view of the order of events. As shown in the scheme of Figure 10, approximately 45 ps before the ζ transition, we detected an increase in the population of ions in the minor groove. Nearly 10 ps later, the twist at the CG step decreases, the minor groove widens and the CH···O interaction begins to form (whenever the presence of a 3′-purine makes this possible). At time 0, the ζ−flip occurs, the CG step transits to the LT state and triggers GA to move to the high slide state, the CH···O contact is formed, with the ions stably located in the minor groove. The reverse analysis, going from tt to the g-g- ζ state, yields a different picture (see details in Supplementary Figure S14). The cations leave the minor groove, starting their migration 250 ps before the ζ transition. Only when the ion occupancy is reduced to its basal value, the twist transit back to the HT state. The dynamics of cation entry and exit from the minor groove are thus substantially different, but play a key role in controlling the kinetics of the CG polymorphism.

To complement this average picture, we computed the ratio between successful/unsuccessful ion-bonding events that lead to HT→LT transitions (see the ‘Materials and Methods’ section for the detailed procedure). The ion density of the ACGA (with K^+^) and GCGA (with Na^+^) tetranucleotides were divided into inner (inside the grooves) and outer regions, into minor and major grooves and into the three steps that define the corresponding tetranucleotide. We found that the ions in the inner region of the minor groove for the CG step have the strongest effect. In the case of K+, they increase the probability of having BII/BII by a factor of 3 (from 0.27 to 0.80), meaning that when there is an ion present in that region of the CG step there is an 80% probability of BII/BII and LT. In the case of Na+, the presence of an ion in the inner minor groove increases the probability of BII/BII by a factor of 2. Ions in the outer minor or major groove of the CG step disfavor significantly the BII/BII state, while the ions at the adjacent steps, in the inner or outer regions, have only weak effects.

## CONCLUSION

We have carried out studies of the unique polymorphism of CG base pair step at an unprecedented level of detail. We have found that the HT/LT conformational transition is the result of a complex choreography of changes. Evidence from very extensive MD simulations indicates that the entrance of cations into the CG minor groove initiates the twist transition. This transition in turn involves BI↔BII changes in the phosphodiester backbone of the 3′-side of the CG step and transitions in the slide of neighboring GR steps. The HT↔LT equilibrium is strongly dependent on the sequence context, and this is found to be linked to the appearance of C8H8···O3′ interactions that stabilize the LT state for certain tetranucleotide sequences. Consequently, CG steps have unique conformational properties that can be finely tuned by the sequence environment. This work confirms and explains the link between polymorphism and cation dynamics, previously described in only a few unrelated experimental (9,10) and theoretical studies (11,18). Ions play a significant role in a concerted and synchronized conformational choreography, controlling not only the thermodynamics, but also the kinetics, of the transitions.

The unique conformational properties of the CG step may be part of the explanation of its significant underrepresentation within the human genome (Supplementary Figure S15). This observation has traditionally been explained by the tendency of CG steps to be methylated, which favors cytosine to thymine mutations as the result of deamination (23). However, such an explanation is not complete, since cytosine underrepresentation also occurs in genomes where this nucleobase is not methylated (*S. Cerevisiae, C. Elegans* and *D. Melanogaster*, see Supplementary Figure S16). It seems then that the unique conformational properties of CG steps may have an impact on their genomic frequency, which may well prefer less conformationally polymorphic steps. In support of this idea, it is remarkable that the most polymorphic TCGA tetranucleotide is the least abundant one in the entire human genome (Supplementary Figure S17). Beyond this general observation, the CG unique physical properties might also be important within specific genomic regions, notably in regulatory regions where many proteins need to recognize and bind to specific sites, sometimes inducing significant deformations. In fact, CG steps turn out to be especially abundant in such regions (Supplementary Figure S18). The fact that protein binding is often coupled to BI-BII transitions (76,88), and that CG steps can also define specific nucleosome arrangement, which are moreover switchable upon methylation (89,90) is compatible with the idea that the unique physical properties of CG may be undesirable in large portions of DNA, but very useful in others.

Lastly, the spontaneous twist polymorphism of CG step may also explain its extreme prevalence in intercalation complexes (11,91–93). To investigate this point we analyzed 74 DNA X-ray structures containing small intercalators (see ‘Materials and Methods’ section). Some of the complexes sample the HT and other the LT conformation. The CG motif is largely preferred over other steps (in 74% of all the intercalation complexes), and curiously two trinucleotides are prevalent in the set of complexes with CG intercalation: CGA (49% of the cases), characterized by high flexibility and the tendency for LT/BII state; and the CGT (37% of the cases), a rigid trinucleotide, which populates mostly the HT/BI state. A crystallographic study of the intercalation of two well-known anticancer drugs into the CG step of CGT and CGA sequences suggested an interesting sequence dependence of the binding (27): The complexes have tighter binding to the CGA trinucleotide due to the formation of inter- and intra-molecular hydrogen bonds that were not observed with the CGT sequence. Although it is clear that further investigation is needed to understand these sequence-dependent binding preferences, it is not unreasonable to think that the CGA trinucleotide is preferred due to its higher flexibility (linked to its ability to form extra hydrogen bonds), and due to its possibility of existing in two clearly distinct conformational states (HT/BI and LT/BII), supporting the conformational selection paradigm.

## SUPPLEMENTARY DATA

Supplementary Data are available at NAR Online.

SUPPLEMENTARY DATA
