# Supplementary material for: Unraveling the sequence-dependent polymorphic behavior of d(CpG) steps in B-DNA
Source: Nucleic Acids Res. 2014 Sep 15;42(18):11304–20. doi: 10.1093/nar/gku809 (PMC4191396; doi:10.1093/nar/gku809)
Supplement: SUPPLEMENTARY DATA [file supp_42_18_11304__index.html]

Unraveling the sequence-dependent polymorphic behavior of d(CpG) steps in B-DNA — Unraveling the sequence-dependent polymorphic behavior of d(CpG) steps in B-DNA — SUPPLEMENTARY DATA 

# Unraveling the sequence-dependent polymorphic behavior of d(CpG) steps in B-DNA

## SUPPLEMENTARY DATA

**Files in this Data Supplement:**

- SUPPLEMENTARY DATA
