# Supplementary material for: Unraveling the sequence-dependent polymorphic behavior of d(CpG) steps in B-DNA
Source: Nucleic Acids Res. 2014 Sep 15;42(18):11304–20. doi: 10.1093/nar/gku809 (PMC4191396; doi:10.1093/nar/gku809)
Supplement: SUPPLEMENTARY DATA [file supp_gku809_nar-01564-f-2014-File002.pdf]

Unraveling the sequence-dependent polymorphic behavior of d(CpG) steps in B-DNA

P. D. Dans[1], I. Faustino[1], F. Battistini[1], K. Zakrzewska[2], R. Lavery[2], M. Orozco[1,3,*]

[1] Joint BSC-CRG-IRB Research Program in Computational Biology, Institute for Research in Biomedicine (IRB Barcelona), Baldiri Reixac 10, Barcelona 08028, Spain.
[2] Bases Moléculaires et Structurales des Systèmes Infectieux, Univ. Lyon I/CNRS UMR 5086, IBCP, 7 Passage du Vercors, Lyon 69367, France.
[3] Departament de Bioquimica, Facultat de Biologia, Avgda Diagonal 647, Barcelona 08028, Spain.

[*] To whom correspondence should be addressed: Prof. Modesto Orozco, Tel: +34 934037155, Fax: +34 934037157, Email: modesto.orozco@irbbarcelona.org.

Supplementary Information

SUPPLEMENTARY TABLES

Table S1. Properties computed for sodium and potassium used in the correlation analysis.

	Potassium			Sodium		
	$k_{twist\text{-}twist}$ [a]	Avg minw [b]	% of BII	$k_{twist\text{-}twist}$	Avg minw	% of BII
ACGA	0.0165	7.32	22	0.0199	7.20	22
ACGC	0.0202	6.42	20	0.0227	6.90	12
ACGG	0.0197	7.26	18	0.0222	7.17	16
ACGT	0.0283	6.17	3	0.0254	6.08	5
CCGA	0.0160	7.87	38	0.0157	7.88	44
CCGC	0.0186	7.66	30	0.0193	7.39	28
CCGG	0.0174	7.91	29	0.0170	7.79	42
GCGA	0.0166	7.66	46	0.0164	7.57	43
GCGC	0.0178	7.60	34	0.0205	7.23	27
TCGA	0.0131	7.89	57	0.0132	7.91	54

[a] Refers to the twist-twist force constant of the CG step in kcal mol^{-1} deg^{-2} [b] Refers to the averaged minor groove width at the CG step in angstroms.

Table S2. Coefficient of determination for the regression model computed for all possible pairs of properties.

	R^2	
	Potassium	Sodium
Cation occupancy vs Weighted average twist	0.95	0.82
Twist-twist force constant vs Weighted average twist	0.80	0.97
BII proportion vs Weighted average twist	0.98	0.99
Minor groove width vs Weighted average twist	0.72	0.90
Cation occupancy vs Twist-twist force constant	0.73	0.77
BII proportion vs Minor groove width	0.62	0.85
Minor groove width vs Twist-twist force constant	0.71	0.87
Cation occupancy vs BII proportion	0.96	0.85

Table S3. CH···O hydrogen bond parameters and density on the bond critical point computed at the MP2(FC)/6-31G(d,p) level.

	Strand	Distance[a]	Angle[b]	ρ (a.u.)	$\nabla^2\rho$ (a.u.)
CC**GA**	Watson	3.2	128.2	0.011	0.039
**CC**GA	Crick	3.2	148.6	0.014	0.046
CC**GG**	Watson	3.2	138.0	0.013	0.043
**CC**GG	Crick	3.3	147.0	0.012	0.037
TC**GA**[c]	Watson	3.0 ± 0.1	136.0 ± 7.4	0.018 ± 0.001	0.059 ± 0.004
**TC**GA[c]	Crick	3.1 ± 0.1	135.1 ± 8.3	0.018 ± 0.001	0.054 ± 0.004

Reference values from ref. 84.

System	H-bond type	Distance	Angle	ρ (a.u.)	$\nabla^2\rho$ (a.u.)
A-U base pair	C2H2···O2	3.6	---	0.006	0.021
A-U base pair	N3H3···N1	2.8	---	0.043	0.100
A-U base pair	N6H6···O4	3.0	---	0.025	0.071
U-U base pair	C5H5···O2	3.3	---	0.016	0.047
U-U base pair	N3H3···O4	2.9	---	0.029	0.084

[a] Hydrogen bond donor-acceptor distance in angstroms. [b] Angle between C8, H8 and O3' in degrees. [c] Average and standard deviation computed over 5 structures chosen along the trajectory.

Table S4. Thermodynamics of the ζ (bp$_{(i+1)}$ in the 3' direction) states and associated average twist for potassium.

	State relative free energy (kcal mol^{-1})				Associated average twist (degrees)			
	g-g-	tg-	g-t	tt	g-g-	tg-	g-t	tt
ACGA	0.0	0.5	2.0	1.1	34.4	26.4	26.6	16.1
ACGC	0.0	1.2	0.9	1.0	35.4	29.2	27.4	17.6
ACGG	0.0	1.2	1.4	1.0	34.5	28.0	26.2	16.9
ACGT	0.0	2.4	1.9	3.2	36.3	27.5	28.4	14.2
CCGA	0.0	0.1	1.2	0.4	32.8	25.0	25.7	16.9
CCGC	0.0	1.0	0.6	0.8	32.9	26.2	25.2	17.4
CCGG	0.0	0.8	0.8	0.7	32.9	25.3	25.6	17.5
GCGA	0.0	0.2	1.0	0.1	33.0	25.7	25.3	17.4
GCGC	0.0	0.8	0.7	0.5	33.1	25.6	25.6	17.7
TCGA	0.0	0.6	0.5	-0.3	32.6	24.2	24.1	15.4

Table S5. Thermodynamics of the ζ states in $bp_{(i)}$ and (associated average twist in degrees).

	First component (C6-G19) State relative free energy (kcal mol^{-1})		Second component (G7-C18) State relative free energy (kcal mol^{-1})	
		K^+Cl^-		
	g-g-	g-t	g-g-	tg-
ACGA	0.0 (31.8)	1.2 (17.9)	0.0 (33.9)	0.4 (23.1)
ACGC	0.0 (34.3)	0.6 (22.4)	0.0 (33.6)	0.8 (21.5)
ACGG	0.0 (33.3)	0.8 (19.7)	0.0 (33.4)	0.8 (20.4)
ACGT	0.0 (35.7)	1.9 (25.7)	0.0 (35.5)	2.3 (24.7)
CCGA	0.0 (29.1)	0.7 (18.5)	0.0 (31.8)	-0.1 (21.7)
CCGC	0.0 (31.7)	0.4 (21.6)	0.0 (30.7)	0.7 (20.5)
CCGG	0.0 (31.2)	0.5 (20.8)	0.0 (31.3)	0.5 (20.9)
GCGA	0.0 (29.9)	0.3 (18.7)	0.0 (31.4)	-0.2 (20.7)
GCGC	0.0 (31.3)	0.3 (20.7)	0.0 (31.2)	0.3 (20.6)
TCGA	0.0 (30.2)	-0.3 (17.4)	0.0 (29.5)	-0.2 (17.0)
		Na^+Cl^-		
	g-g-	g-t	g-g-	tg-
ACGA	0.0 (32.4)	1.3 (19.7)	0.0 (34.5)	0.4 (24.6)
ACGC	0.0 (33.7)	1.5 (22.5)	0.0 (34.6)	1.1 (25.2)
ACGG	0.0 (33.5)	1.2 (22.2)	0.0 (34.0)	0.8 (24.6)
ACGT	0.0 (36.2)	1.8 (24.7)	0.0 (36.2)	1.7 (25.5)
CCGA	0.0 (29.4)	0.3 (18.6)	0.0 (31.6)	-0.1 (20.7)
CCGC	0.0 (32.6)	0.5 (23.3)	0.0 (32.0)	0.6 (22.8)
CCGG	0.0 (30.4)	0.3 (20.8)	0.0 (30.8)	0.1 (21.5)
GCGA	0.0 (30.3)	0.5 (19.3)	0.0 (32.1)	-0.1 (21.8)
GCGC	0.0 (32.1)	0.6 (23.4)	0.0 (32.1)	0.6 (23.2)
TCGA	0.0 (30.4)	-0.1 (18.6)	0.0 (30.7)	-0.2 (18.9)
		Drew-Dickerson dodecamer (4 microseconds)		
	First component (C3-G22)		Second component (G4-C21)	
GCGA	0.0 (34.1)	0.7 (20.6)	0.0 (35.5)	0.1 (23.9)

SUPPLEMENTARY FIGURES

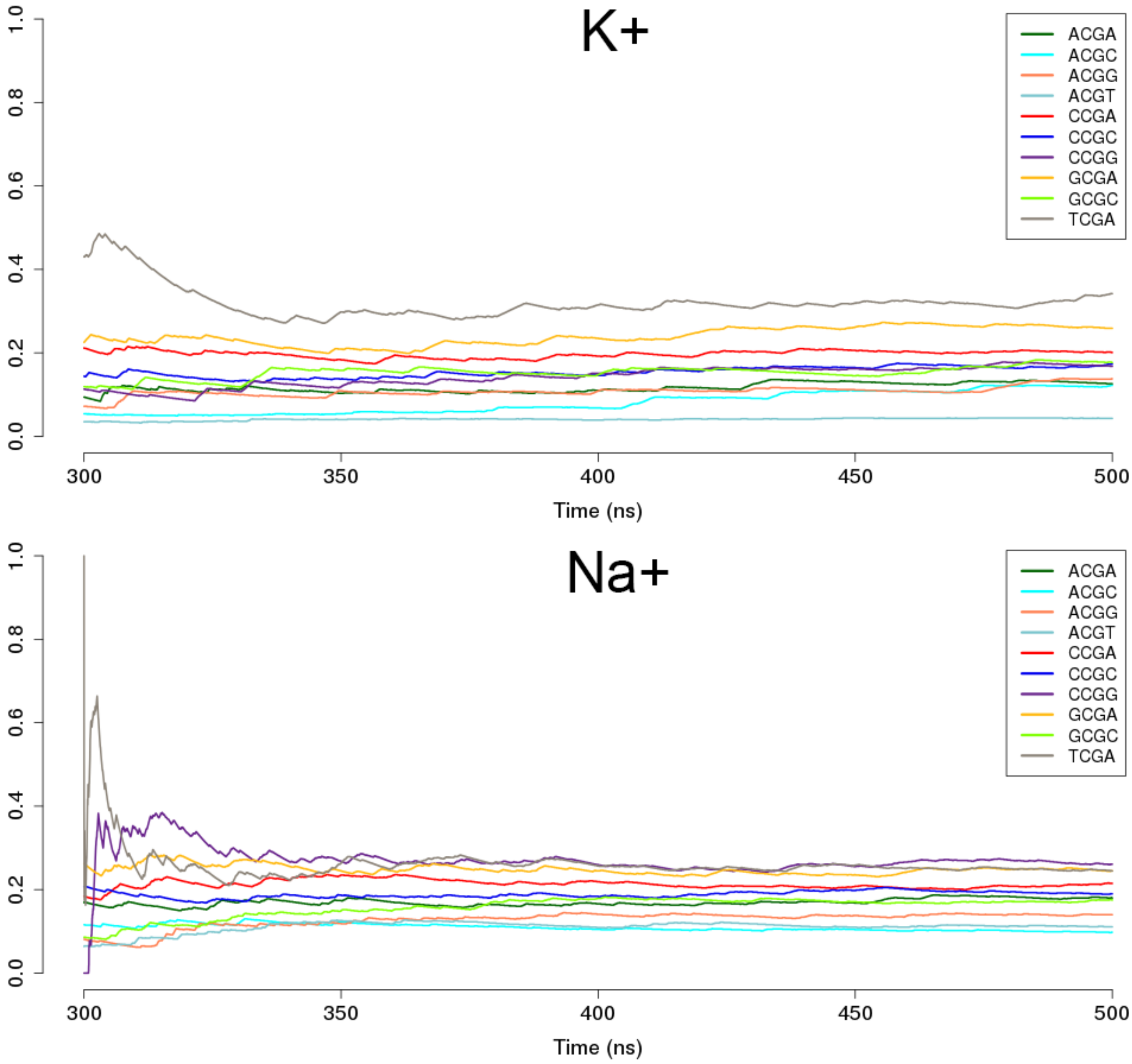

Figure S1. Time-averaged K^+ and Na^+ populations within the DNA minor groove for the 10 unique tetranucleotides with a central CG step, for increasing durations (ns) of the molecular dynamics trajectory.

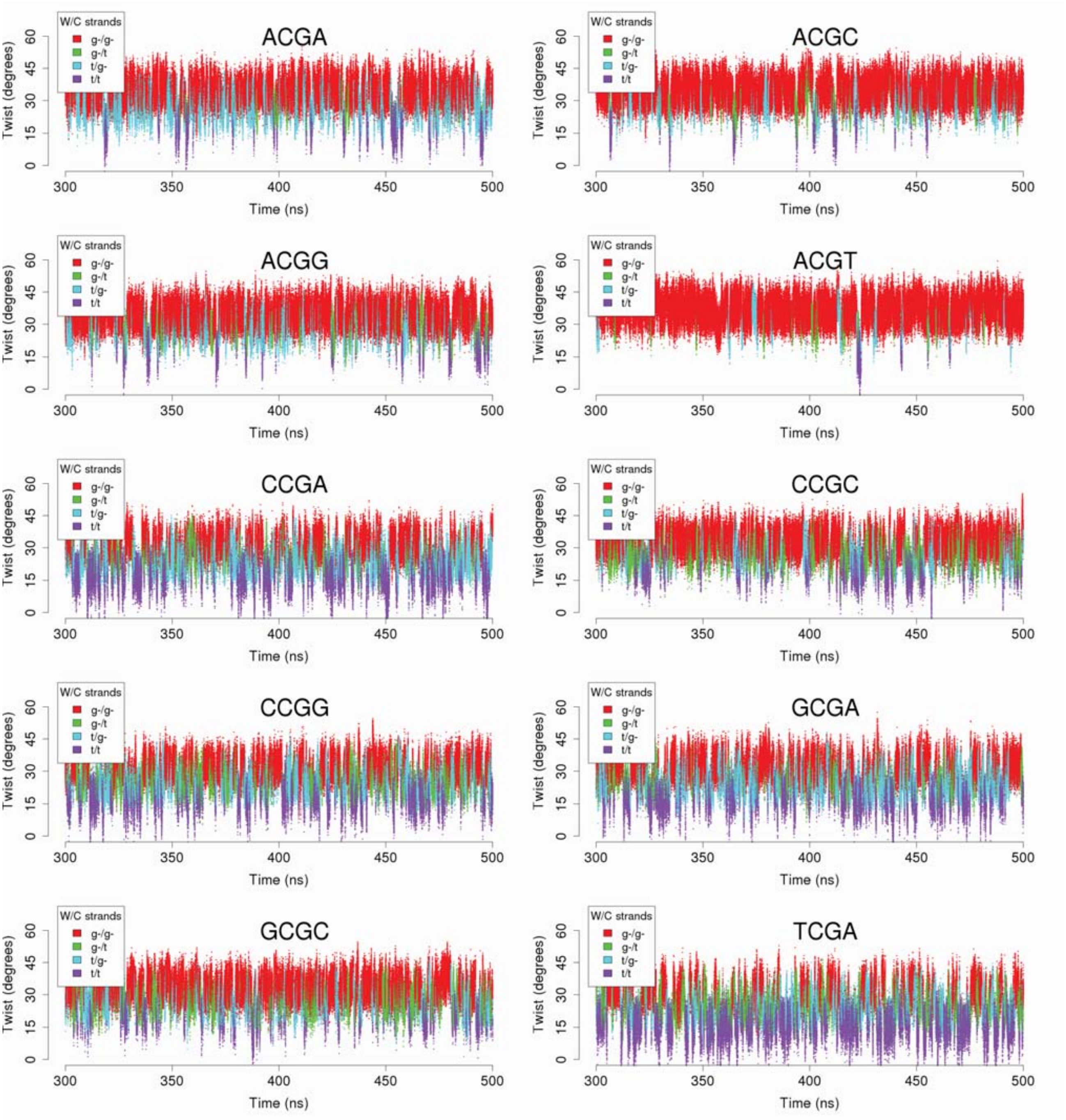

Figure S2. Correlations between twist at the central CG step and the states of the ζ angle at the 3'-side for Na^+Cl^-.

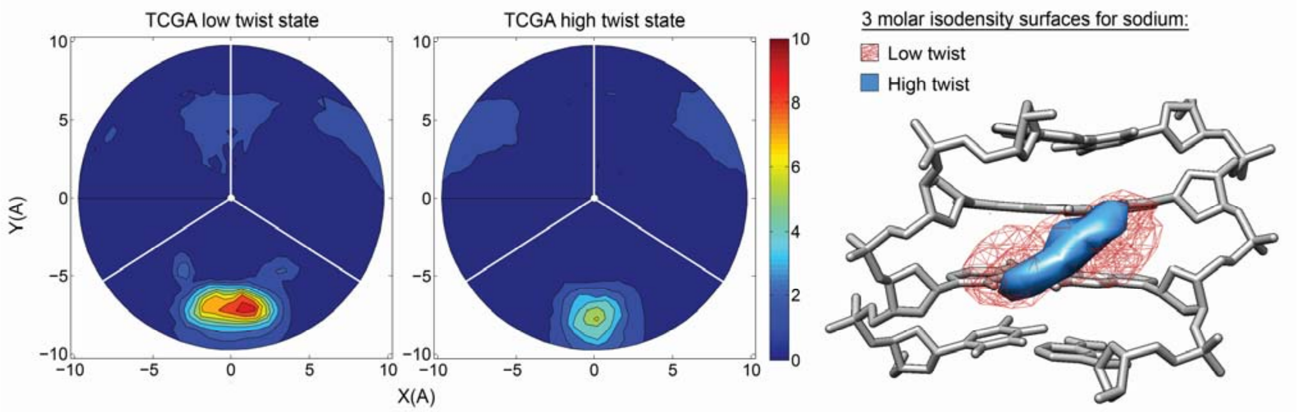

Figure S3. 2-Dimensional Na^+ distributions obtained by filtering the TCGA trajectory according to the twist states of the CG step. Radial-angular plane at the central CG step. The results are plotted as molarities as shown by the color bars, with a blue to red concentration scale that goes from 0 to 10 molar. The plots show the minor groove limits as white lines and the center of the major groove as a vertical radial vector. For sake of comparison, the 3-dimensional distribution plots display the same molarity isodensity surface of 3 molar.

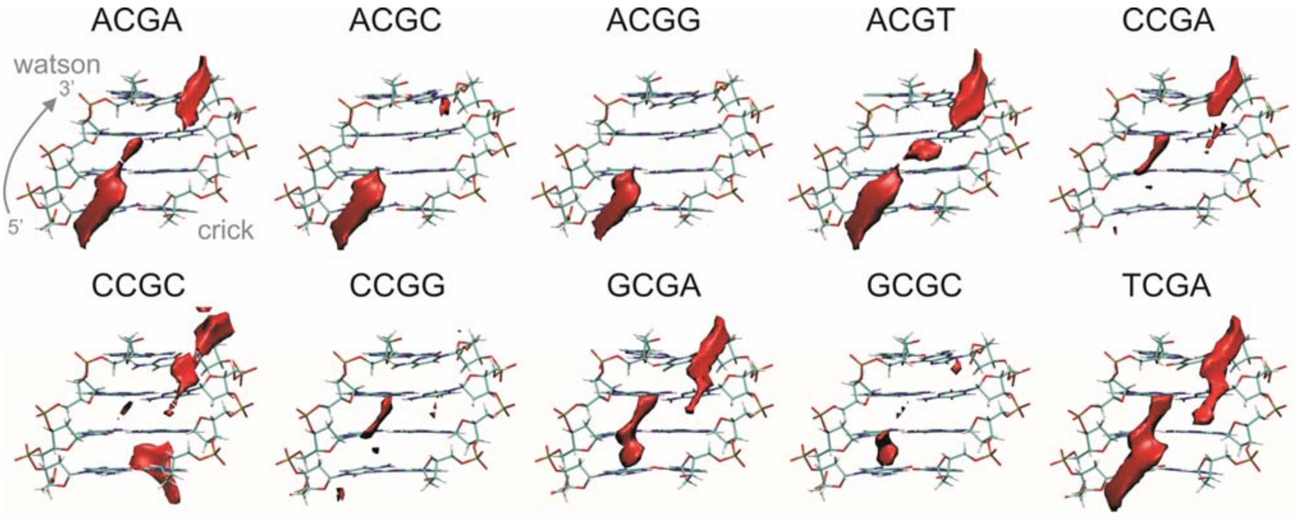

Figure S4. Classical molecular interaction potentials using sodium as a probe. For sake of comparison, all the averaged structure were aligned and the same isosurface of -7.5 kcal mol^{-1} was computed. Similar results were obtained using potassium as a probe (data not shown).

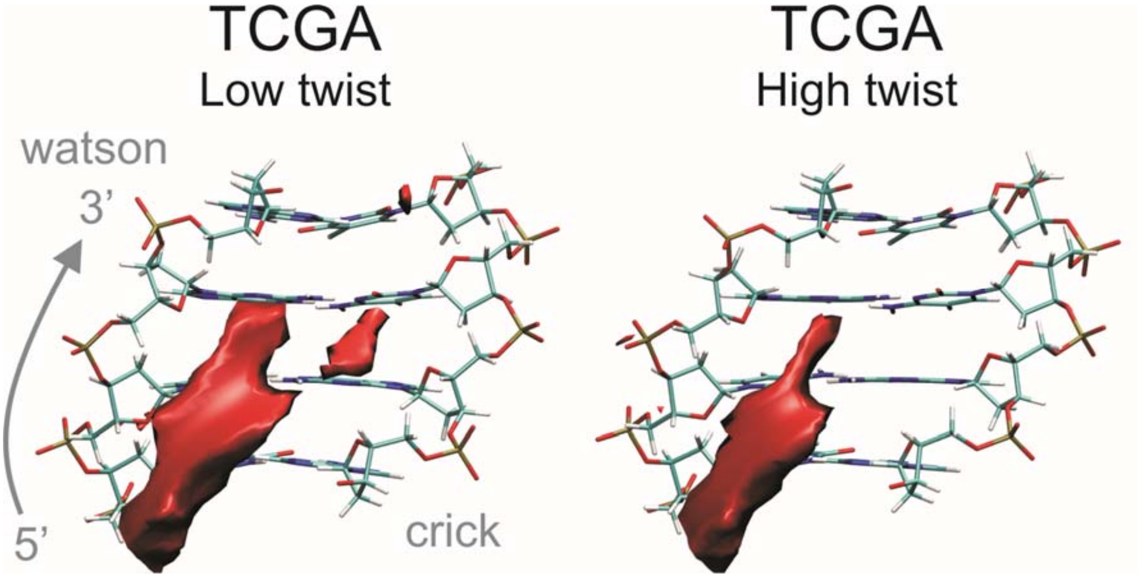

Figure S5. Classical molecular interaction potentials using sodium as a probe. For sake of comparison, the two averaged structures were aligned and the same isosurface of -7.5 kcal mol^{-1} was computed.

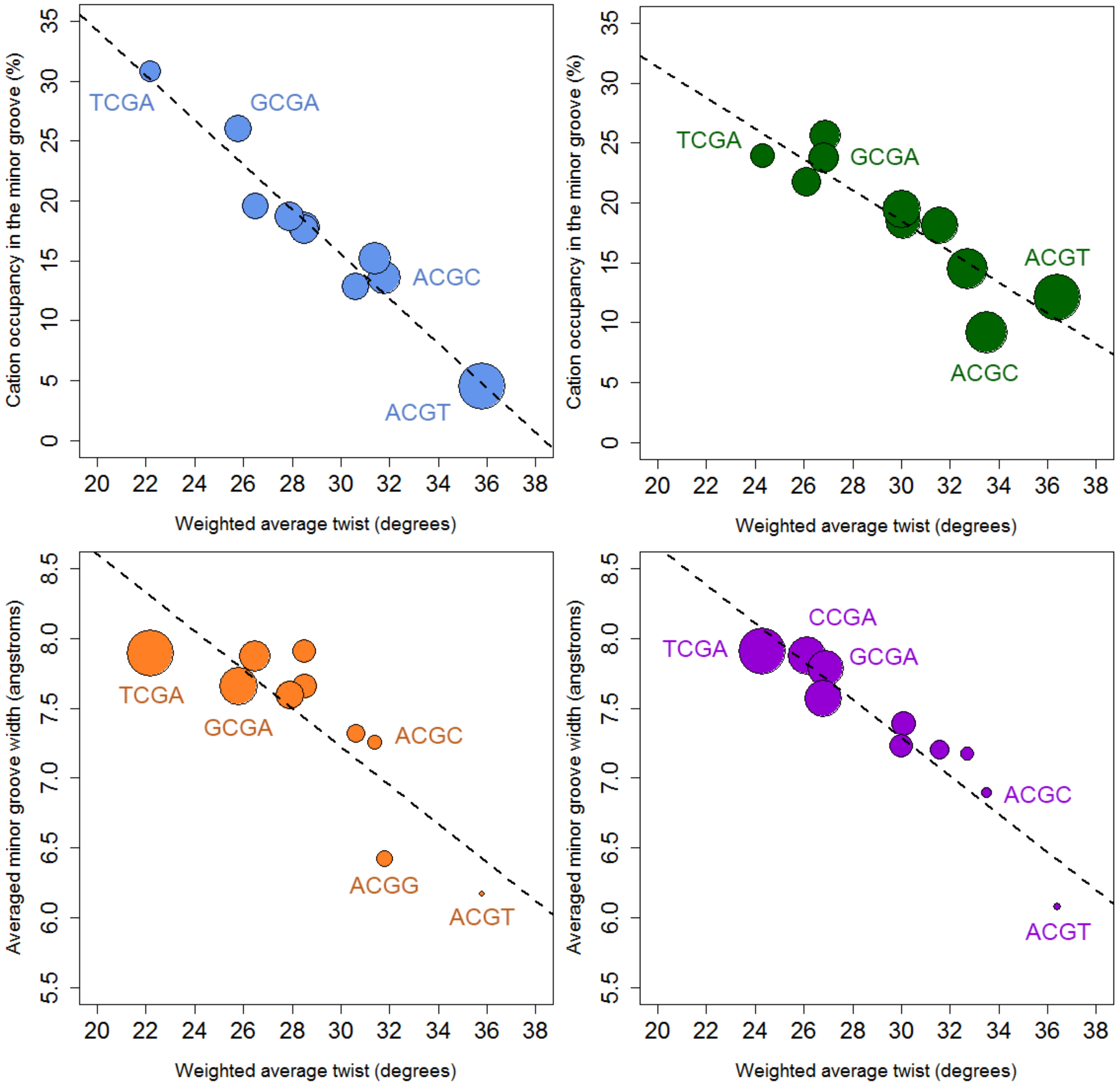

Figure S6. Correlations between the weighted average twist of the CG step, and several relevant properties. The dashed lines represent the fitting with a linear regression model. The size of the points represents the pure twist-twist force constants (upper panels), or the proportion of the BII substates (bottom panels); smaller points means more flexible tetranucleotides, and less BII substates, respectively.

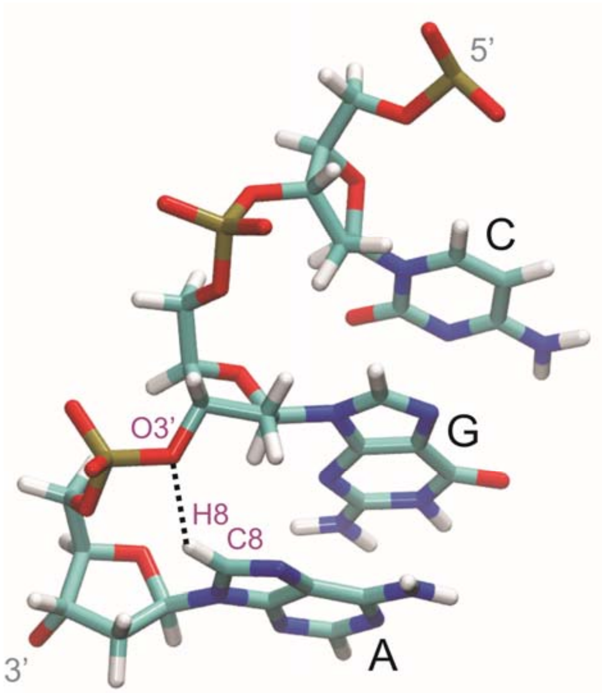

Figure S7. Representation of the C8H8···O3’ interaction in the CGA trinucleotide.

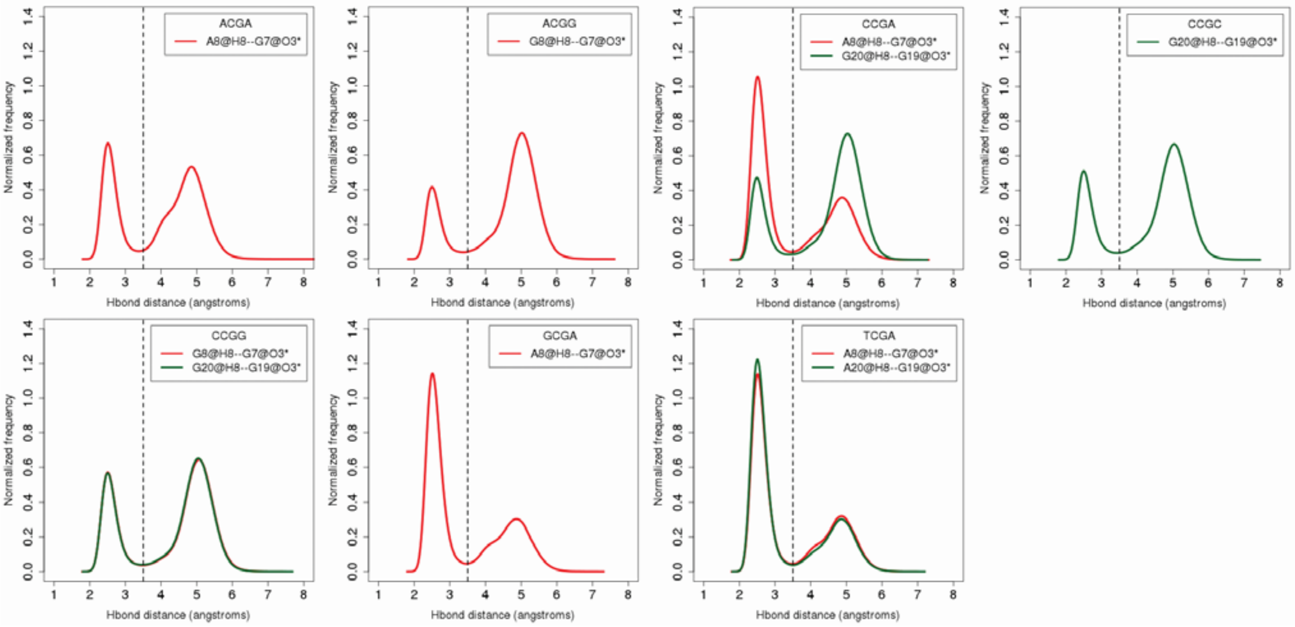

Figure S8. Distribution of the distances of the $C8H8_{(i+1)}$-$O3'_{(i)}$ "hydrogen bonds", for all the cases simulated with K^+Cl^-. Exactly the same results are found with sodium (data not shown). To follow the coupling between the twist at the CG step and the formation of the CH···O interaction, we define the bond as formed when the acceptor-donor distance was below 3.5 Å (vertical black dashed lines).

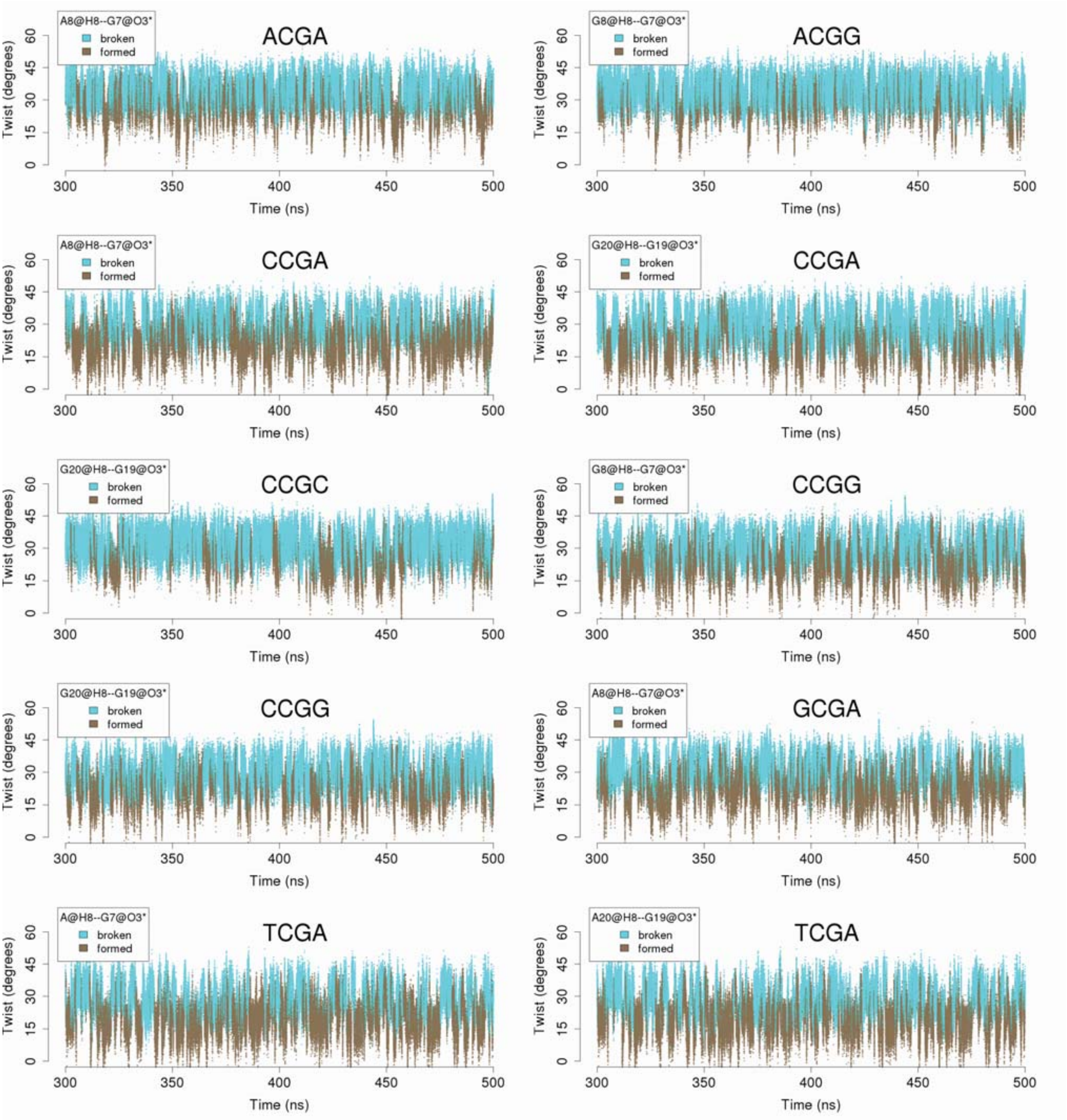

Figure S9. Time evolution of the twist at the CG step and the formation of the intra-molecular CH···O interaction. Results for the 10 possible tetranucleotides simulated in $Na^{+}Cl^{-}$.

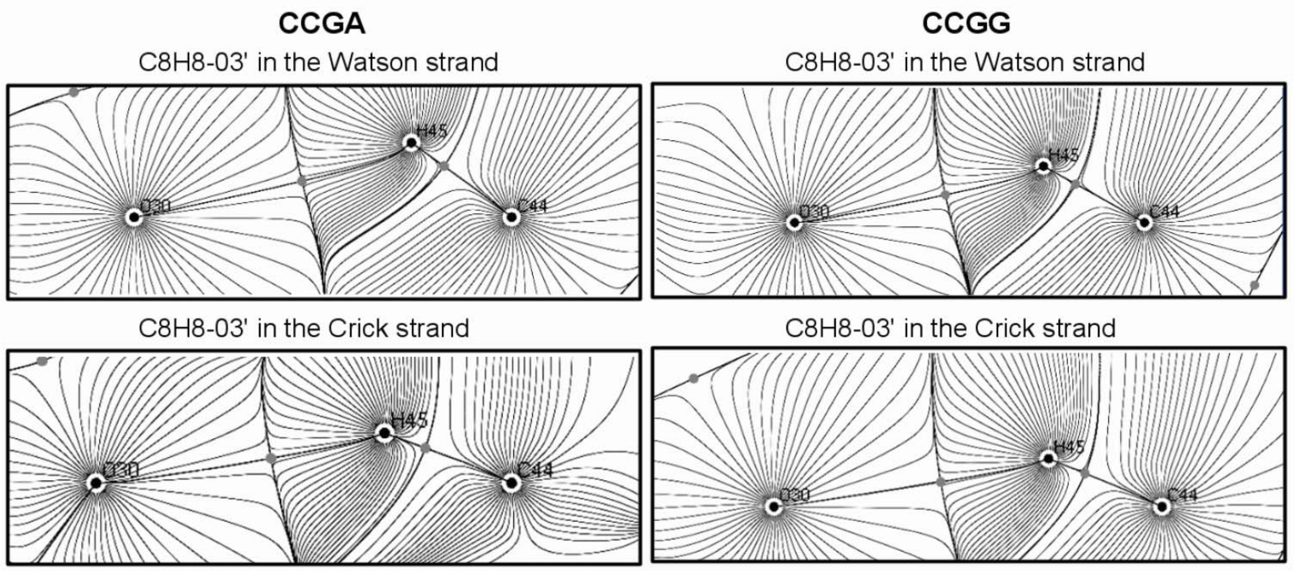

Figure S10. Hydrogen bond AIM analysis for the CCGA (left) and CCGG (right) tetranucleotides in the BII/BII conformation. The atoms labelled as C44, H45 and O30 represent the C8, H8 and O3' atoms of the flanking purine. The bond critical points are evidenced by gray dots. The nuclear critical points (located at the position of the nuclei), the basin paths, and the gradient field are depicted in solid black. The bond paths, defined by the chosen 2-dimensional projection (plane), are also shown as black lines.

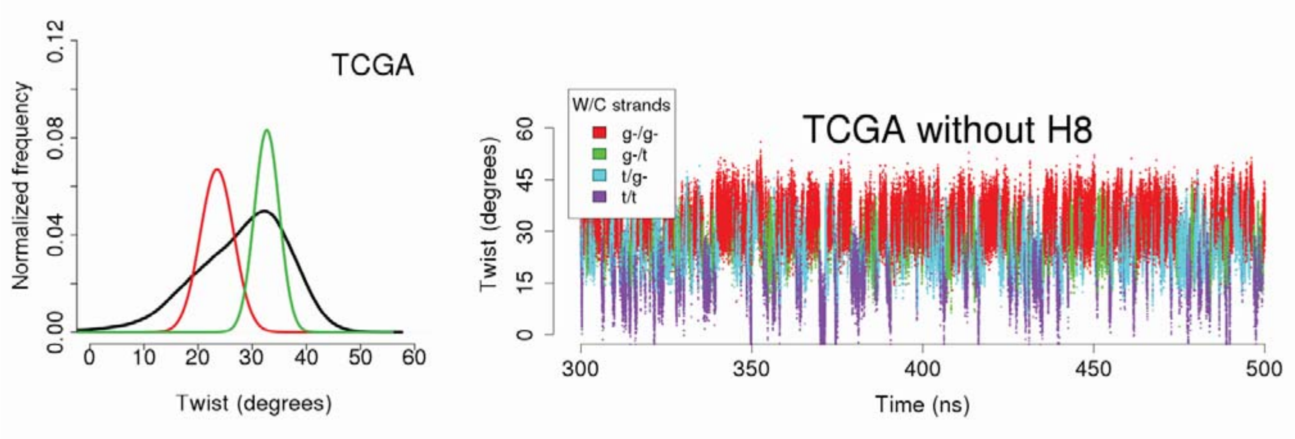

Figure S11. Twist distribution and correlation between twist and the possible states of the ζ angles. The observed distribution is depicted in black and normal components obtained with BIC in red (low twist component), and green (high twist component) respectively. Correlation between the twist of the central CG step and the four states of the ζ angle at the 3'-side are shown at the right of the distributions.

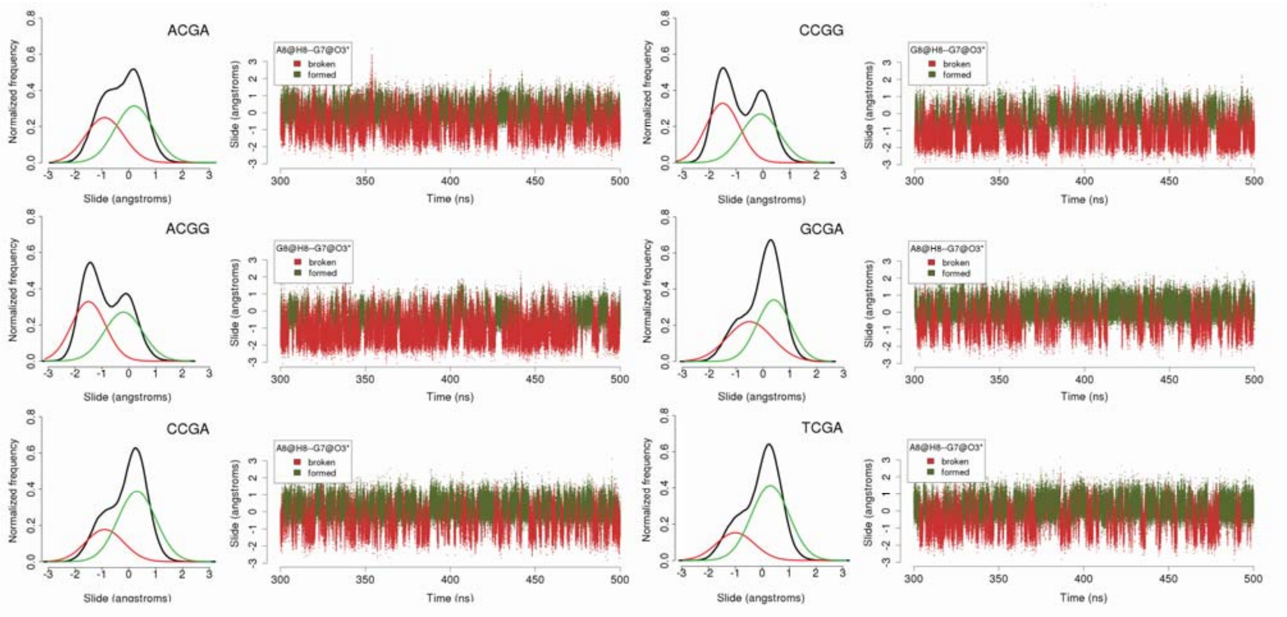

Figure S12. Slide distribution at the next-neighbor GR step and the formation of the intra-molecular CH···O contact. The slide distribution at the next-neighbor GR step is shown in black and normal components obtained with BIC in red (low slide component) and green (high slide component) respectively. Correlation between the slide of the GR step and the two possible states of the CH···O interaction (formed/broken) for all the cases with GR steps in the Watson strand are shown at the right of the distributions. For the sake of clarity, only the results with $K^{+}Cl^{-}$ are shown. Equivalent results were found with $Na^{+}Cl^{-}$ (data not shown).

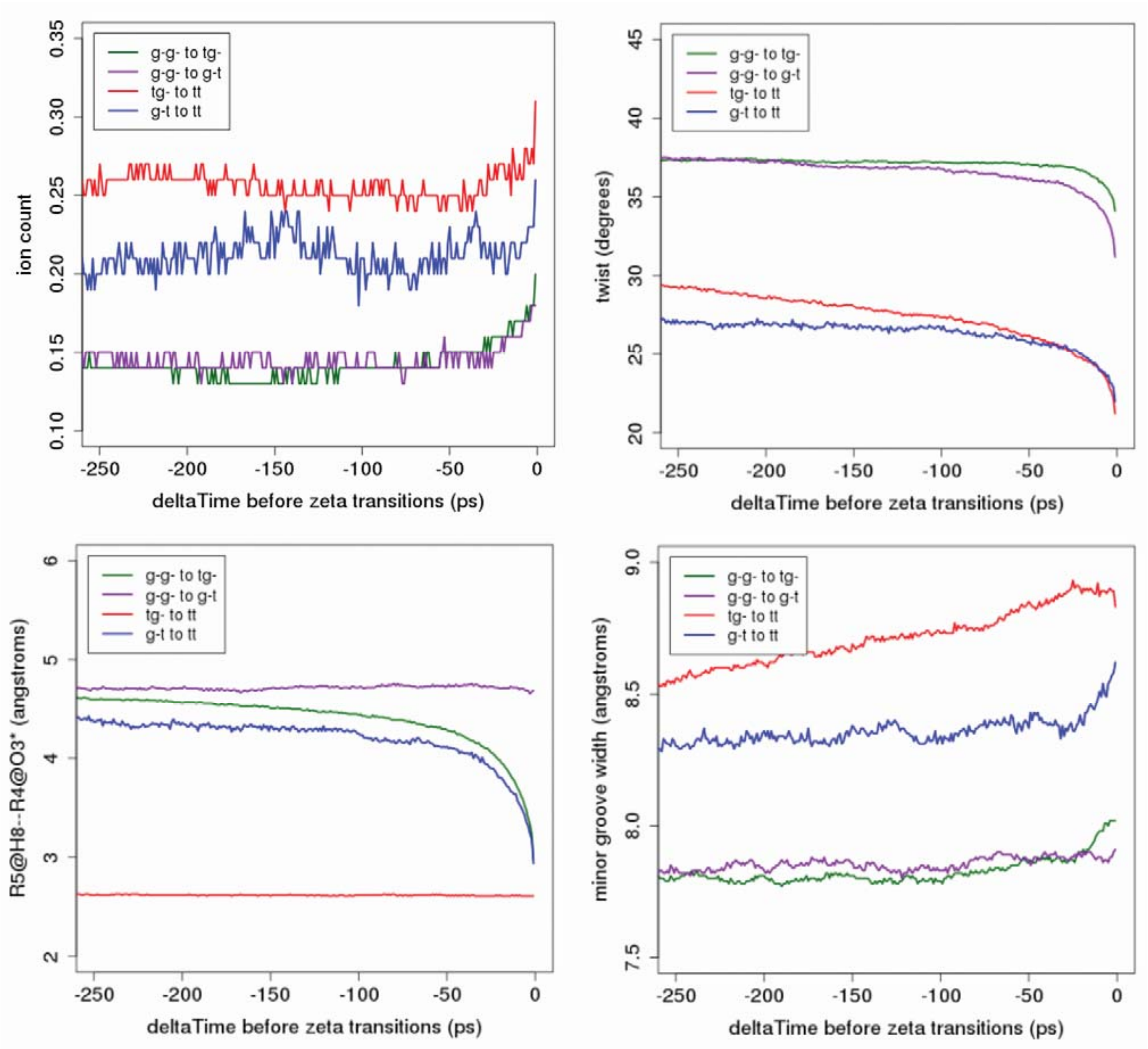

Figure S13. Causality analysis for the ζ transitions of the CG step, from the g-g- to the final tt substate. The sodium occupancy, the twist helical parameter, the intra-molecular hydrogen bond, and the minor groove width were inspected.

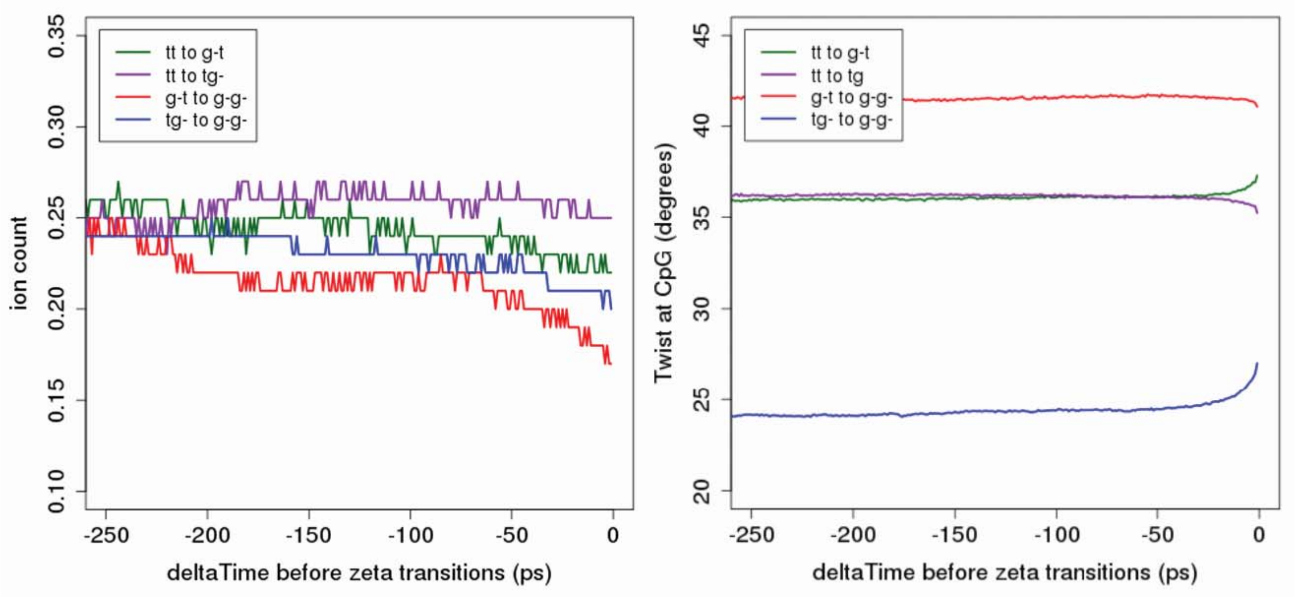

Figure S14. Causality analysis of the CG step, when moving from the tt to the final g-g- substate. The sodium occupancy and the twist helical parameter were inspected.

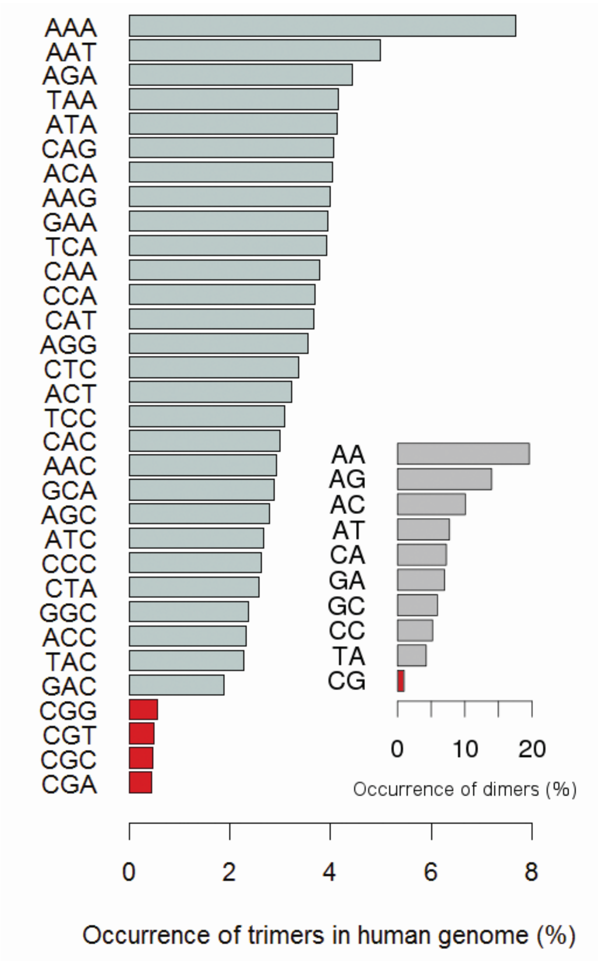

Figure S15. Occurrence of each possible di- and trinucleotides in the human genome.

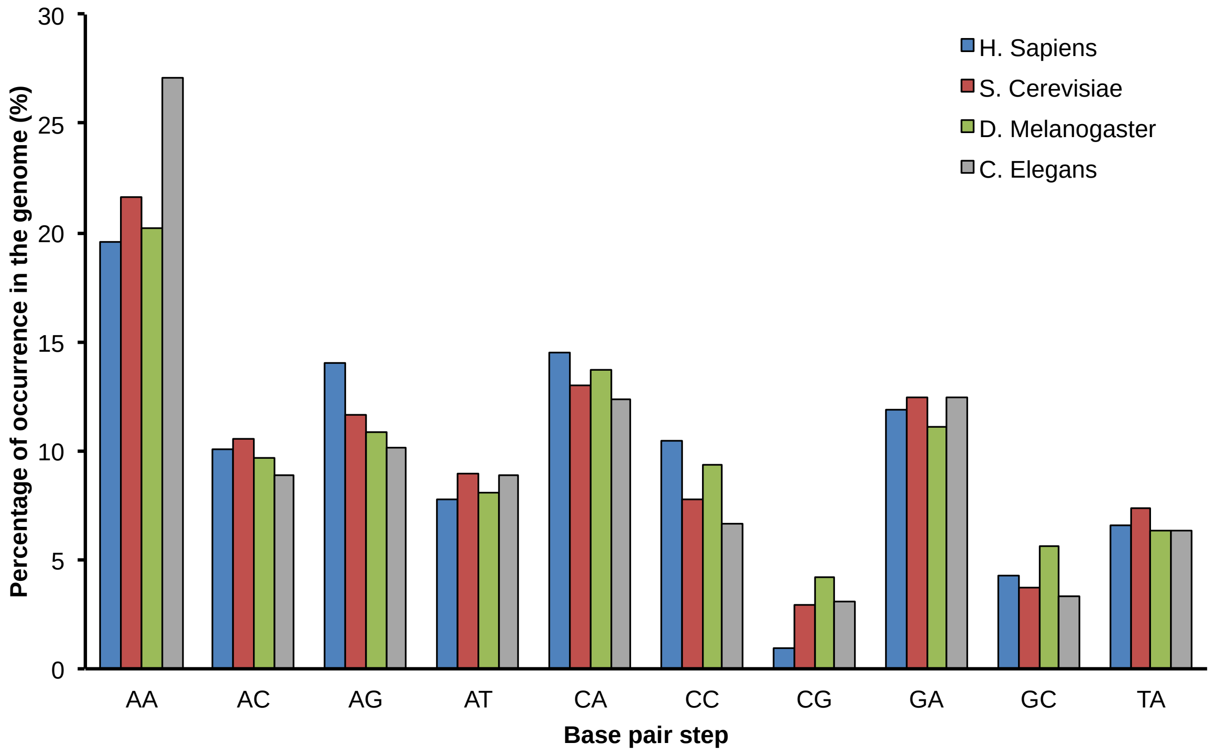

Figure S16. Occurrence of each possible base pair step in different organism, normalized by the length of each genome.

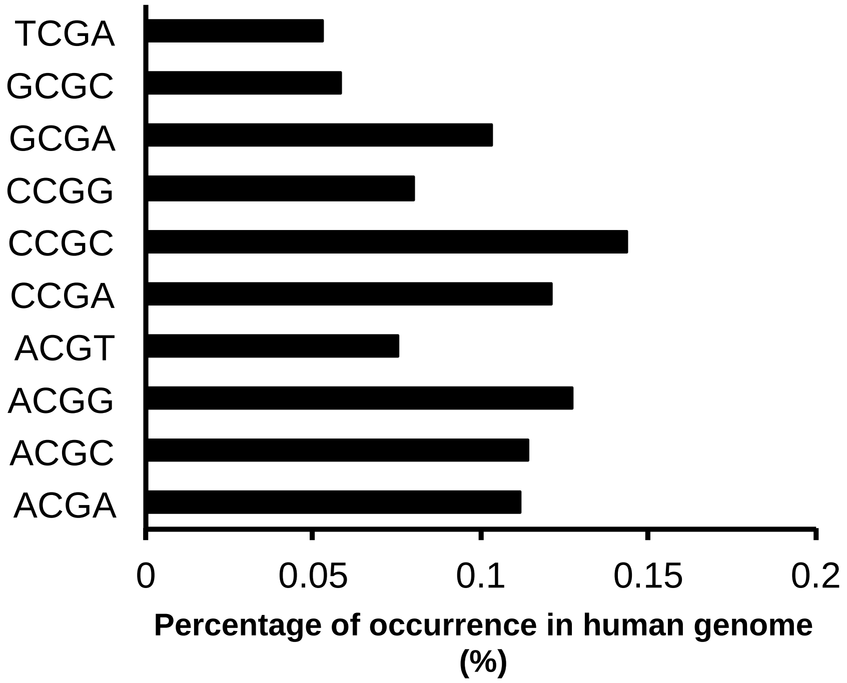

Figure S17. Occurrence of each possible tetranucleotide with central CG step in the human genome.

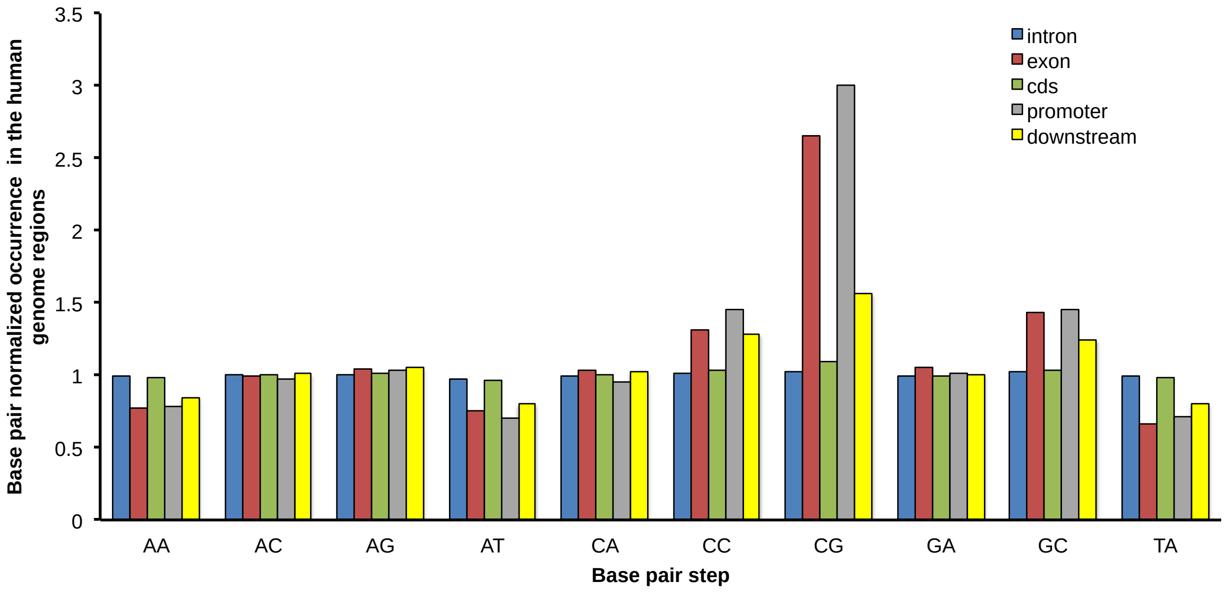

Figure S18. Occurrence of each possible base pair step (%) in different human genome regions, normalized by the length of each region and by the ratio of each base pair step in the overall genome.
